# RNF43 Inactivation Enhances the B‐RAF/MEK Signaling and Creates a Combinatory Therapeutic Target in Cancer Cells

**DOI:** 10.1002/advs.202304820

**Published:** 2024-01-15

**Authors:** Shih‐Han Hsu, Ya‐Li Tsai, Yeng‐Tseng Wang, Che‐Hung Shen, Yu‐Hsuan Hung, Li‐Tzong Chen, Wen‐Chun Hung

**Affiliations:** ^1^ National Institute of Cancer Research National Health Research Institutes Tainan 704 Taiwan; ^2^ Department of Biochemistry College of Medicine Kaohsiung Medical University Kaohsiung 807 Taiwan; ^3^ Department of Medical Research Kaohsiung Medical University Hospital Kaohsiung Medical University Kaohsiung 807 Taiwan; ^4^ Division of Gastroenterology Department of Internal Medicine Kaohsiung Medical University Hospital Kaohsiung 804 Taiwan; ^5^ Faculty of Medicine College of Medicine Kaohsiung Medical University Kaohsiung 807 Taiwan; ^6^ Department of Pharmacy, College of Pharmacy Kaohsiung Medical University Hospital Kaohsiung 807 Taiwan; ^7^ Department of Biological Science and Technology National Yang Ming Chiao Tong University Hsinchu 300 Taiwan

**Keywords:** B‐RAF, mitogen‐activated protein kinase kinase (MEK), RING finger protein 43 (RNF43), Ubiquitin ligases, Wingless and Int‐1 (WNT)

## Abstract

RING finger 43 (RNF43), a RING‐type E3 ubiquitin ligase, is a key regulator of WNT signaling and is mutated in 6–10% of pancreatic tumors. However, RNF43‐mediated effects remain unclear, as only a few in vivo substrates of RNF43 are identified. Here, it is found that RNF43‐mutated pancreatic cancer cells exhibit elevated B‐RAF/MEK activity and are highly sensitive to MEK inhibitors. The depletion of RNF43 in normal pancreatic ductal cells also enhances MEK activation, suggesting that it is a physiologically regulated process. It is confirmed that RNF43 ubiquitinates B‐RAF at K499 to promote proteasome‐dependent degradation, resulting in reduced MEK activity and proliferative ability in cancer cells. In addition, phosphorylation of B‐RAF at T491 suppresses B‐RAF ubiquitination by decreasing the interaction between RNF43 and B‐RAF. Mutations at K499 in B‐RAF are identified in various cancer types. MEK and WNT inhibitors synergistically suppress the growth of RNF43‐mutated pancreatic cancer cells in vitro and in vivo. Collectively, the research reveals a novel mechanism by which RNF43 inhibits B‐RAF/MEK signaling to suppress tumor growth and provide a new strategy for the treatment of RNF43‐inactivated pancreatic cancer.

## Graphical abstract

It is demonstrated that RNF43 ubiquitinates B‐RAF at K499, promoting its degradation. This ubiquitination process is negatively regulated by phosphorylation at T491. Inactivation of RNF43 enhances sensitivity to MEK inhibitors and creates synergy between drugs targeting the WNT and MEK pathways in various RNF43‐mutated cancers.

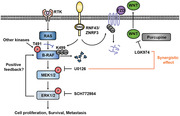

## 1 Introduction

Ubiquitination is a highly ordered post‐translational modification that regulates numerous aspects of cellular functions by controlling the homeostasis of intracellular proteins. Conjugation of ubiquitin (Ub) to target proteins is catalyzed by Ub‐activating (E1), Ub‐conjugating (E2), Ub‐ligating (E3), and deubiquitinating enzymes.^[^ ^1^ ^]^ Compared to the limited numbers of E1 and E2 enzymes, more than 600 genes in the human genome are predicted to encode E3 ligases, indicating the importance of target recognition.

Ring finger 43 (RNF43), an E3 ligase with a RING finger motif for protein‐protein interaction, was originally identified as a differentially expressed gene in human colorectal tumors.^[^ ^2^ ^]^ RNF43 and its homolog ZNRF3 were first identified as negative regulators of WNT pathway by Hao et al.^[^ ^3^ ^]^ Although several RNF43‐interacting proteins have been identified in proteomics studies, only a few of them have been confirmed as in vivo substrates of this E3 ligase.^[^ ^4^ ^]^ Currently, the most well‐characterized physiological substrates of RNF43 are frizzled (FZD) proteins, the cognate receptors of WNT ligands. A mechanistic study revealed that RNF43 directly ubiquitinates FZD receptors and targets them to lysosomes for degradation.^[^ ^5^ ^]^

B‐RAF is a key upstream activator of mitogen‐activated protein kinase kinase (MEK) and extracellular signal‐regulated kinase (ERK) signaling that stimulates cell proliferation.^[^ ^6^ ^]^ Gain of function mutations in the *B‐Raf* gene are frequently observed in melanoma, colon cancer, and lung cancer.^[^ ^7^ ^]^ Numerous studies suggest that B‐RAF is a critical driver of oncogenesis.^[^ ^8^ ^]^ In addition, the location of mutations in the B‐RAF protein substantial affects RAS dependency, dimerization, and drug response.^[^ ^9^ ^]^

Here, we discovered that RNF43 inactivation results in enhanced B‐RAF/MEK signaling and increased MEK inhibitor sensitivity. Molecular characterization revealed that RNF43 induces ubiquitination and degradation of B‐RAF to restrain MEK activation in cells. In addition, B‐RAF ubiquitination by RNF43 is negatively regulated by phosphorylation. Collectively, we identified B‐RAF as a substrate for RNF43 and clarified how RNF43 inactivation promotes B‐RAF activation to accelerate tumorigenesis.

## 2 Results

### 2.1 The B‐RAF/MEK Signaling is Negatively Regulated by RNF43 in Pancreatic Cancer Cells

**Figure 1:**
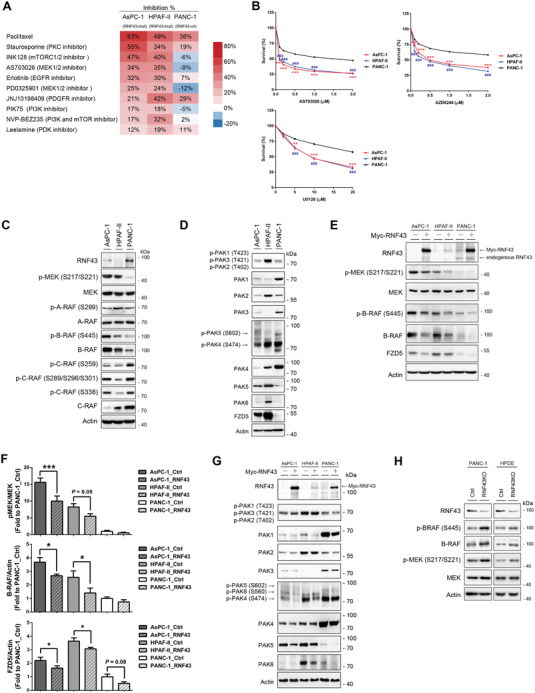
B‐RAF and MEK are constitutively activated in *RNF43*‐mutated pancreatic cancer Cells. A) Kinase screening in *RNF43*‐mutated AsPC‐1 and HPAF‐II and *RNF43*‐wild‐type PANC‐1 cells. Top10 kinases with the strongest inhibition are shown (*n* = 3). B) Sensitivity of AsPC‐1, HPAF‐II, and PANC‐1 cells to MEK inhibitors AS703026, AZD6244, and U0126 (*n* = 9). Data are presented as mean ± SEM. *, AsPC‐1 compared with PANC‐1; **P < 0.01, ***P < 0.001 by ANOVA. ^#^, HPAF‐II compared with PANC‐1; ^###^ *P* < 0.001 by ANOVA. C,D) Protein expression and activation of the RAF/MEK signaling molecules (C) and the PAK family (D) in pancreatic cells with wild‐type or mutant RNF43. FZD5: Frizzled5 protein. E–G) The ectopic expression of RNF43 on expression and activation of B‐RAF/MEK signaling molecules (E) and the PAK family (G) in pancreatic cancer cells. F) Quantification results of MEK activity (*n* = 6), B‐RAF levels (*n* = 4), and FZD5 levels (*n* = 6). Data are presented as mean ± SEM. *P < 0.05, **P < 0.01, ***P < 0.001 by ANOVA. H) CRISPR/Cas9 targeting of RNF43 in PANC‐1 cells and HPDE cells induced activation of the B‐RAF/MEK signaling pathway. RNF43KO: RNF43 knockout cells. (Related Figures S1 and S2, Table S1, Supporting Information).

*RNF43* is mutated in various solid tumors, including pancreatic cancer. Using a genome‐wide clustered regularly interspaced palindromic repeat (CRISPR) approach, a previous study demonstrated that the WNT receptor FZD5 signaling circuit is vulnerable in *RNF43*‐mutated pancreatic cancer.^[^ ^10^ ^]^ To identify the altered signaling pathways and druggable targets in *RNF43*‐mutated cancer cells, we performed kinase inhibitor library screening in *RNF43*‐mutated pancreatic cancer cells (AsPC‐1 and HPAF‐II) and *RNF43*‐wild‐type PANC‐1 cells. Our data showed significantly higher sensitivities of *RNF43*‐mutated cells to the MEK inhibitors AS703026 and PD0325901 (**Figure** **1**A). Similar results were obtained using additional MEK inhibitors (AZD6244 and U0126), suggesting that this was not a drug‐specific phenomenon (Figure 1B; and Table S1, Supporting Information). Increased sensitivity to MEK inhibitors was confirmed in *RNF43*‐mutated melanoma and colorectal cancer cells (Table S1, Supporting Information). *RNF43*‐mutated pancreatic cancer cells exhibited enhanced MEK activity (Figure 1C). It is well‐established that MEK undergoes phosphorylation and activation, facilitated by members of the RAF family (A‐, B‐, and C‐RAF) and the p21‐activated kinase (PAK) family.^[^ ^11^ ^]^ Among these proteins, only the activities of B‐RAF and PAK5 were consistent with MEK activation in all three cell lines (Figure 1C,D), suggesting that B‐RAF and PAK5 are potential upstream kinases that control MEK activation in *RNF43*‐mutated cancer cells. FZD5 protein levels were extremely low in *RNF43*‐wild‐type PANC1 cells, consistent with the finding that FZD5 is an in vivo substrate of RNF43 (Figure 1D).

The ectopic expression of RNF43 decreased MEK activity and FZD5 expression in AsPC‐1 and HPAF‐II cells, but not in PANC‐1 cells (Figure 1E,F). The enzymatic activity and protein levels of B‐RAF were attenuated in AsPC‐1 and HPAF‐II cells overexpressing RNF43, indicating that RNF43 may downregulate B‐RAF protein (Figure 1E,F). Moreover, overexpression of RNF43 decreased B‐RAF protein levels in melanoma and colorectal cancer cells containing mutated RNF43 or B‐RAF (Figure S1, Supporting Information). While the protein level of PAK5 was affected similarly to that of B‐RAF by RNF43 (Figure 1G), no interaction between RNF43 and PAK5 was detected (Figure S2A, Supporting Information). Our findings exclude the possibility that RNF43 modulates MEK activation by interacting with PAK5. Depletion of RNF43 in PANC‐1 and normal human pancreatic ductal epithelial (HPDE) cells increased the protein levels of B‐RAF and the activities of B‐RAF and MEK, suggesting that this regulatory mechanism exists in both cancer and normal cells (Figure 1H). Our results suggest that RNF43 is a negative regulator of B‐RAF. Mutations in the *RNF43* gene are observed in neoplastic cysts of the pancreas, indicating that it is an early event in pancreatic tumorigenesis and is crucial for tumor formation.^[^ ^12^ ^]^ As the *K‐RAS* mutation also occurs in the pre‐cancerous lesions of pancreas, we hypothesized that RNF43 inactivation may help the cells escape from *K‐RAS*‐induced senescence. Indeed, RNF43 depletion counteracted senescence in *K‐RAS*‐transfected cells (Figure S3, Supporting Information).

### 2.2 RNF43 Ubiquitinates B‐RAF at K499 to Promotes Its Proteasome‐Dependent Proteolysis

**Figure 2:**
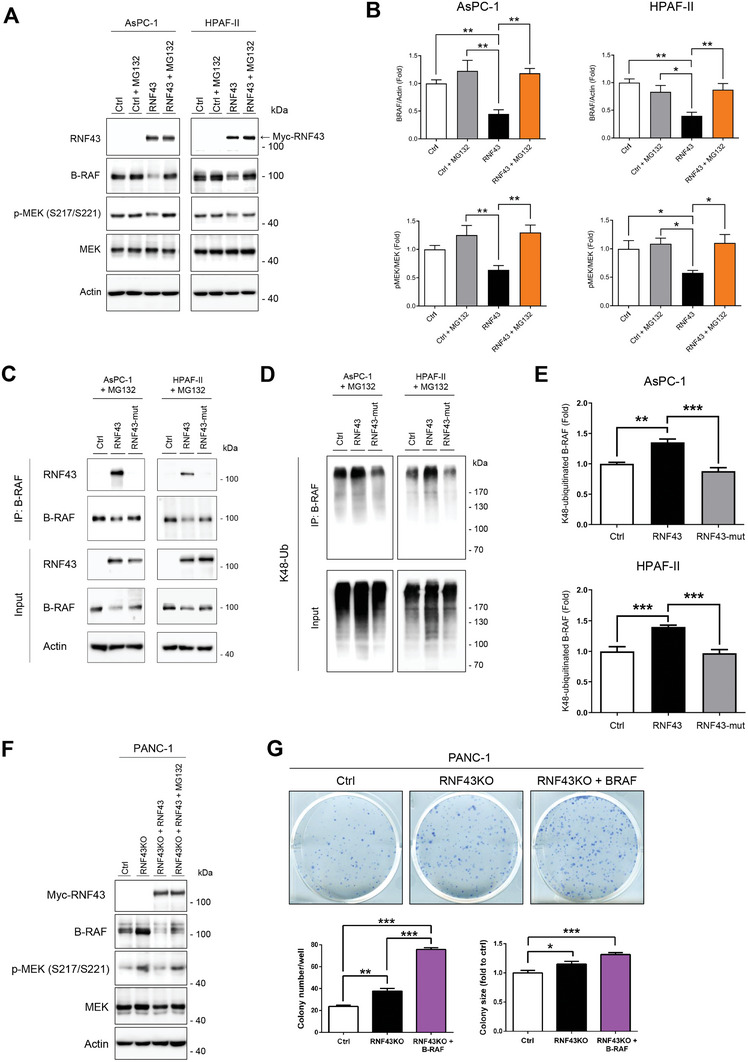
RNF43 facilitates the ubiquitination‐dependent proteolysis of B‐RAF. A,B) Effect of the proteasome inhibitor MG132 (30 µm for 3 h) on B‐RAF and MEK in *RNF43‐*mutated AsPC‐1 and HPAF‐II cells overexpressing RNF43. B) Quantification of B‐RAF levels and MEK activities in AsPC‐1 (*n* = 3) and HPAF‐II (*n* = 3) cells overexpressing RNF43 with or without MG132 additions. Data are presented as mean ± SEM. *P < 0.05, **P < 0.01 by ANOVA. C–E) AsPC‐1 and HPAF‐II cells were transfected with the control vector, wild‐type RNF43, or C290S/C298S mutated RNF43 plasmid (RNF43‐mut). Protein expression of RNF43, B‐RAF, and MEK (C) were examined. The K48‐ubiquitination of B‐RAF (D and E) was investigated by immunoprecipitation and immunoblotting assays. E) Quantification of K48‐ubiquitinated B‐RAF levels immunoprecipitated with B‐RAF antibody in AsPC‐1 (*n* = 3) and HPAF‐II (*n* = 4) cells transfected with wild‐type RNF43 or mutated RNF43. Data are presented as mean ± SEM. **P < 0.01, ***P < 0.001 by ANOVA. F) RNF43KO PANC‐1 cells were transfected with wild‐type RNF43. MG132 effect on B‐RAF and MEK was examined with western blotting. G) The colony‐forming abilities of RNF43KO PANC‐1 cells with or without overexpressing B‐RAF. (Related Figure S2, Supporting Information).

When AsPC‐1 and HPAF‐II cells were treated with the proteasome inhibitor MG132, the downregulation of B‐RAF protein by RNF43 overexpression was reversed (**Figure** **2**A,B). In addition, the inactivation of RNF43 ligase by mutations at C290 and C298 in the catalytic domain (RNF43‐mut) substantially impaired the reduction of B‐RAF (Figure 2C). Immunoprecipitation and immunoblotting assays showed increased B‐RAF ubiquitination by the overexpression of wild‐type RNF43, but not the inactive RNF43 mutant, confirming the requirement of ligase activity for RNF43 to K48‐ubiquitinate B‐RAF (Figure 2D,E). Ectopically expressed Myc‐tagged RNF43 pulled down B‐RAF in AsPC‐1 cells in a reverse immunoprecipitation assay, suggesting a direct interaction between B‐RAF and RNF43 (Figure S2A, Supporting Information). CRISPR/Cas9 gene targeting was performed to knockout RNF43 (RNF43KO) in PANC‐1 cells. RNF43 depletion increased B‐RAF protein levels and MEK activity (Figure 2F). Re‐expression of RNF43 in RNF43KO cells induced B‐RAF downregulation and reduced MEK activity, which was consistent with the results in AsPC‐1 and HPAF‐II cells (Figure 2A–C,F). In addition, the colony‐forming ability of PANC‐1 cells was increased in RNF43KO cells and further enhanced by B‐RAF overexpression (Figure 2G).

### 2.3 K499 Mutation Identified in Cancer Patients Increases the B‐RAF Protein Stability and Cell Proliferation

**Figure 3:**
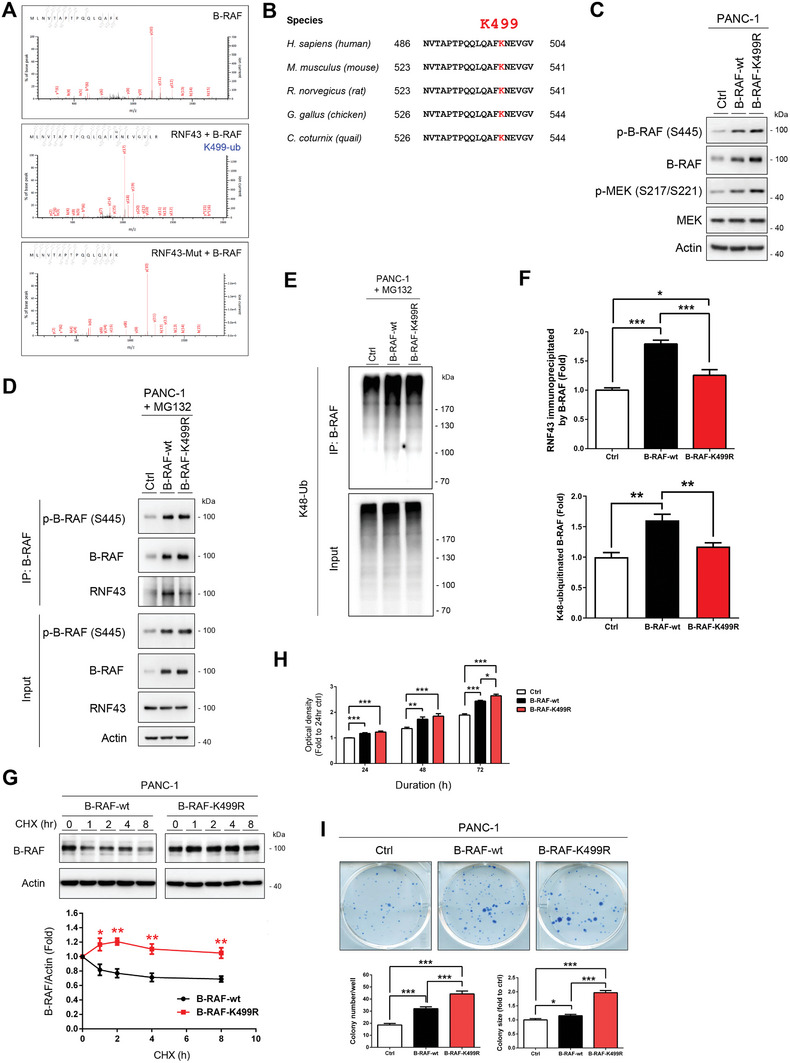
K499R mutation in B‐RAF results in elevated protein stability and proliferating ability of PANC‐1 cells. A) Mass spectrometry analysis identified ubiquitination at K499 in B‐RAF protein by RNF43. B) Alignment of the 486–504 region of human B‐RAF with homologous sequences from various species, highlighting the position of K499. C–I) PANC‐1 cells were transfected with control, wild‐type B‐RAF, or K499R mutant plasmids. The protein levels and activities of indicated proteins (C) were studied. After the treatment with MG132, the kinase activity (D) and K48‐ubiquitination of B‐RAF (E) were determined. F) Quantification of RNF43 levels (*n* = 6) and K48‐ubiquitinated B‐RAF levels (*n* = 3) immunoprecipitated with B‐RAF antibody. Data are presented as mean ± SEM. *P < 0.05, ***P < 0.001 by ANOVA. G) After the treatment with cycloheximide (20 µg/mL), the protein stabilities of wild‐type B‐RAF and K499R mutant were compared (*n* = 4). Data are presented as mean ± SEM. *P < 0.05, **P < 0.01 by T‐test. Cellular proliferations (H, *n* = 12) and the numbers and sizes of colonies (I, *n* = 4) were measured. Data are presented as mean ± SEM. *P < 0.05, **P < 0.01, ***P < 0.001 by ANOVA. (Related Figure S4 and Table S2, Supporting Information).

Using a ubiquitination assay followed by mass spectrometry, we identified K499 as a specific site in the B‐RAF protein ubiquitinated by RNF43 (**Figure** **3**A). K499 is located within a highly conserved region across various species (Figure 3B), implying that ubiquitination at this site may have critical biological functions. Analysis of clinical databases, including cBioPortal (https://www.cbioportal.org/) and the Catalogue Of Somatic Mutations In Cancer (COSMIC, https://cancer.sanger.ac.uk/cosmic) identified cancer patients with K499 mutations in B‐RAF (Figure S4 and Table S2, Supporting Information). To test the effect of the K499 mutation on B‐RAF, we generated a B‐RAF K499R mutant. Accumulated B‐RAF and higher B‐RAF and MEK activities were detected in PANC‐1 cells expressing B‐RAF‐K499R (Figure 3C). Decreased interaction between RNF43 and B‐RAF‐K499R compared to B‐RAF‐wt. was observed (Figure 3D,F). The K48‐ubiquitination of B‐RAF was reduced in PANC‐1 cells transfected with the K499R mutant compared to cells transfected with wild‐type B‐RAF (Figure 3E,F). A pulse‐chase assay using cycloheximide, a protein synthesis inhibitor, revealed the enhanced stability of the K499R mutant (Figure 3G). Moreover, overexpression of the K499R mutant considerably increased proliferation and colony formation in PANC‐1 cells compared to that in wild‐type B‐RAF (Figure 3H,I), suggesting that the K499 mutation increases B‐RAF stability and downstream signaling to promote cell growth.

### 2.4 Phosphorylation at T491 in B‐RAF Inhibits the RNF43‐Mediated B‐RAF Ubiquitination

Next, we addressed whether phosphorylation affects the K499 ubiquitination in B‐RAF by RNF43; since phosphorylation‐dependent ubiquitination is a general mechanism for controlling protein homeostasis. The first candidate residue was S445 because constitutive phosphorylation at S445 enhances the basal activity of B‐RAF.^[^ ^13^ ^]^ In addition, this residue is conserved in B‐RAF proteins of various species (Figure S5A, Supporting Information). However, the growth‐promoting effect, colony‐forming activity, and MEK activation were similar between the wild‐type B‐RAF and S445A groups (Figure S5B–D, Supporting Information). Additionally, the S445 mutation did not affect B‐RAF protein stability (Figure S5E, Supporting Information). These data excluded the involvement of S445 phosphorylation in the regulation of B‐RAF ubiquitination.

**Figure 4:**
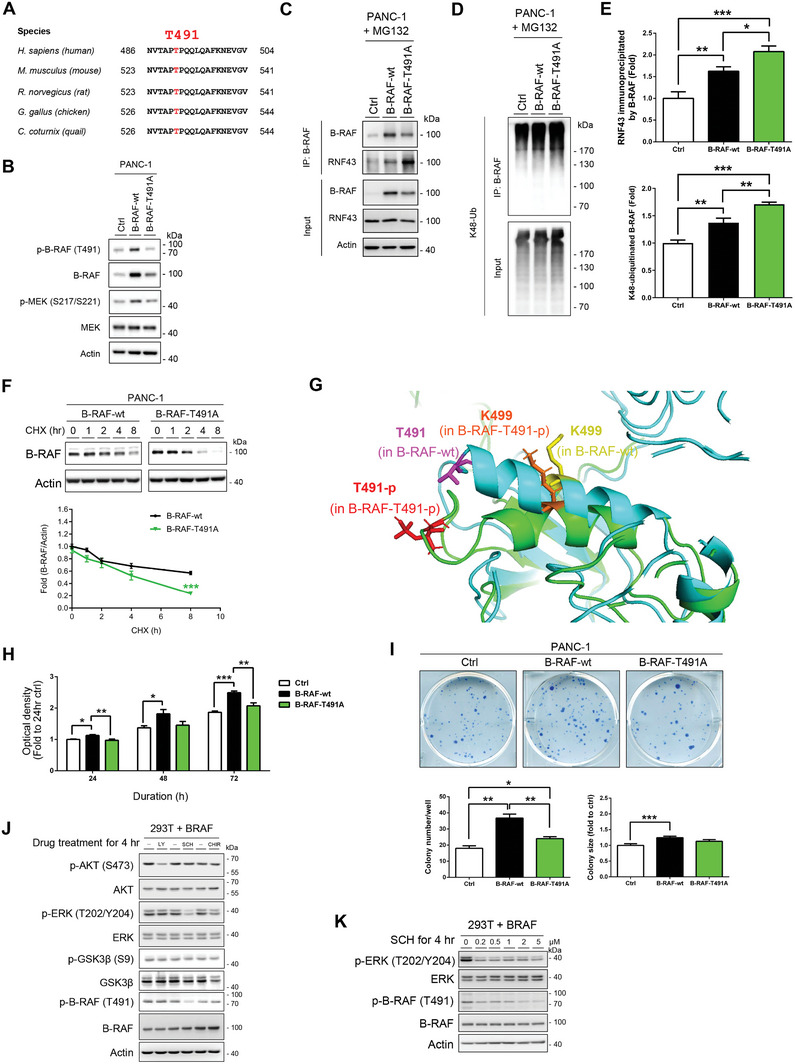
T491A mutation in B‐RAF decreases the protein stability and the downstream signaling of B‐RAF in cancer progression. A) Alignment of the 486–504 region of human B‐RAF with homologous sequences from various species, highlighting the position of T491. B–E) PANC‐1 cells were transfected with control, wild‐type B‐RAF, or T491A mutant plasmids. The protein levels and activities of indicated proteins (B) in the cells were studied. The interaction between RNF43 and B‐RAF (C), and the K48‐ubiquitination of B‐RAF (D) were determined. E) Quantification of RNF43 levels (*n* = 8) and K48‐ubiquitinated B‐RAF levels (*n* = 4) immunoprecipitated with B‐RAF antibody. Data are presented as mean ± SEM. *P < 0.05, **P < 0.01, ***P < 0.001 by ANOVA. F) PANC‐1 cells transfected with wild‐type B‐RAF or T491A mutant plasmids were treated with cycloheximide. Protein stabilities of wild‐type B‐RAF and T491A mutant were compared (*n* = 5). Data are presented as mean ± SEM. ***P < 0.001 by T‐test. G) Phosphorylation at T491 led to a conformational change in the 485–503 region of B‐RAF. H,I) Cellular proliferations (H, *n* = 12) and the numbers and sizes of colonies (I, *n* = 4) of control vector‐, wild‐type B‐RAF‐, and T491A mutant‐transfected cells were measured. Data are presented as mean ± SEM. *P < 0.05, **P < 0.01, ***P < 0.001 by ANOVA. J) HEK293T cells were transfected with wild‐type B‐RAF expression plasmids and treated with inhibitors against PI3K/AKT, ERK, or GSK3. The B‐RAF phosphorylation at T491 was investigated. LY: LY294002, PI3K/AKT inhibitor. SCH: SCH772984, ERK inhibitor. CHIR: CHIR99021, GSK3 inhibitor. K) The HEK293T cells transfected with wild‐type B‐RAF plasmids were treated with various concentrations of SCH. The ERK phosphorylation at T202/Y204 and the B‐RAF phosphorylation at T491 was investigated. (Related Figures S5 and S6, Table S3, Supporting Information).

When examining the residues adjacent to K499, a nearby T491 residue caught our attention. This site is located within a highly conserved region of various species (**Figure** **4**A). Additionally, the threonine preceding proline is an essential regulatory motif that can be phosphorylated by various proline‐directed protein kinases to elicit diverse biological processes.^[^ ^14^ ^]^ We generated a phospho‐specific antibody to detect T491 phosphorylation and confirmed that both T491 phosphorylation and MEK activation were substantially reduced in T491A‐transfected cells (Figure 4B). Increased interaction between RNF43 and B‐RAF was observed (Figure 4C,E), along with enhanced K48‐ubiquitination of the T491A mutated B‐RAF compared to wild‐type B‐RAF in transfected cells (Figure 4D,E). The results suggested that T491 phosphorylation negatively regulates RNF43‐mediated B‐RAF ubiquitination. Indeed, the half‐life of the T491A mutant was considerably shorter than that of wild‐type B‐RAF (Figure 4F). Conversely, the phospho‐mimic mutant, T491E, was quite stable (Figure S5F, Supporting Information). Molecular dynamics (MD) simulations were performed to investigate the effect of T491 phosphorylation on B‐RAF protein structure. A pronounced conformational change in the 485–503 region of B‐RAF was observed upon phosphorylation at T491 (Figure 4G; Figure S6A, Supporting Information). We constructed an RNF43 model that mimics the biologically relevant structure (Figure S6B, Supporting Information). MD simulations were performed by replacing Ariadne RBR E3 ubiquitin protein ligase 1 (ARIH1) in the ARIH1‐UbcH7‐Ub complex with RNF43 to construct the 3D structure of the complex (Figure S6C, Supporting Information).^[^ ^15^ ^]^ Phosphorylation at T491 reduced the accessibility of K499 to the RNF43‐containing E3 complex and decreased its affinity for ubiquitin‐binding (Table S3, Supporting Information), consistent with our finding that the T491A‐mutated B‐RAF showed higher affinity for RNF43 (Figure 4C,E). In culture, the T491A mutant did not considerably promote the proliferation of pancreatic cancer cells (Figure 4H). In addition, it only marginally augmented colony formation (Figure 4I). These findings suggest that phosphorylation at T491 attenuates the interaction between B‐RAF and RNF43, offering a molecular basis to elucidate why the T491A mutant is more susceptible to ubiquitination by RNF43. We also attempted to identify the kinases that can phosphorylate T491. Reduced T491 phosphorylation in B‐RAF was observed in cells treated with the ERK1/2 inhibitor (SCH772984), but not with the phosphoinositide 3‐kinase (PI3K)/AKT inhibitor (LY294002) or glycogen synthase kinase‐3 (GSK3) inhibitor (CHIR99021) (Figure 4J). Additionally, SCH772984 inhibited T491 phosphorylation in a dose‐dependent manner (Figure 4K). These data suggest the possibility of a feedback mechanism for ERK in regulating B‐RAF stability.

### 2.5 Inhibition of RNF43‐Mediated B‐RAF Ubiquitination Promotes Tumorigenesis

**Figure 5:**
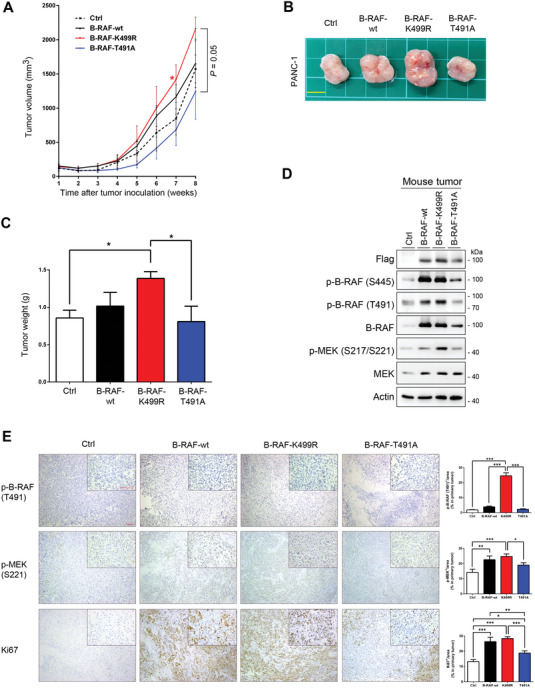
Inhibition of the B‐RAF ubiquitination at K499 promotes tumorigenesis in vivo. Nude mice were subcutaneously injected at the right flank with PANC‐1 cells stably expressing the control vector, wild‐type BRAF, BRAF‐K499R, or BRAF‐T491A (*n* = 4 for each group). A) Alteration in averaged tumor volumes in different experimental groups of mice. Data are presented as mean ± SEM. *Compared with T491A group, *P < 0.05 by ANOVA. B) Typical picture of excised tumor in each group. scale bar: 1 cm. C) Averaged tumor weights of different experimental groups of mice at week 8 after cancer cell injection. Data are presented as mean ± SEM. *P < 0.05 by ANOVA. D) Phosphorylation and protein levels of B‐RAF and MEK in harvested mouse tumors. E) Immunohistochemical staining of p‐B‐RAF (T491), p‐MEK (S221), and Ki67 in mouse tumors. The whole picture is in 100X and the inserted picture is in 400X, scale bar: 100 µm. Data are presented as mean ± SEM. *P < 0.05, **P < 0.01, ***P < 0.001 by ANOVA.

Our results demonstrate that RNF43 ubiquitinated B‐RAF in a phosphorylation‐dependent manner to suppress proliferation and colony formation in cancer cells. We assessed the in vivo tumorigenic activities of various mutants. Nude mice were subcutaneously injected with PANC‐1 cells ectopically expressing wild‐type B‐RAF, K499R, or T491A mutants. Our data showed that tumor growth in the K499R group was substantially increased, whereas it was reduced in the T491A group compared to that in the control group (**Figure** **5**A–C). Moreover, the protein levels and activities of B‐RAF and MEK were elevated in the tumors of the wild‐type and K499R groups, as observed in cultured cancer cells (Figure 5D). Immunohistochemical staining confirmed the enhancement of T491 phosphorylation, MEK activity, and Ki67 expression in the tumors of these two groups, and these elevations were detected in cancer cells but not in stromal cells (Figure 5E). These results suggest that inhibition of RNF43‐mediated B‐RAF ubiquitination at K499 promotes tumor growth.

### 2.6 Synergism of the WNT and MEK Inhibitors in *RNF43*‐Mutated Cancer Cells

**Figure 6:**
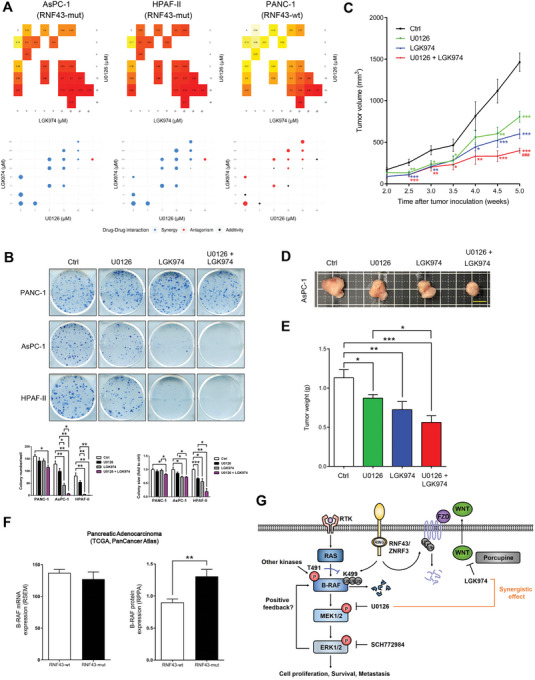
Combination of the WNT and MEK inhibitors showed synergistic effects in *RNF43*‐mutated cancer cells. A) AsPC‐1, HPAF‐II, and PANC‐1 cells were exposed to various concentration of MEK inhibitor U0126 and WNT inhibitor LGK974. Cell viabilities and the combination index (CI) were investigated (*n* = 9). Blue dots indicated synergistic cytotoxic effects, black dots indicated additivities, and red dots in indicated antagonisms. B) Clonogenic assay results showed the effect of combination of U0126 and LGK974 in AsPC‐1, HPAF‐II, and PANC‐1 cells (*n* = 4). Data are presented as mean ± SEM. *P < 0.05, **P < 0.01, ***P < 0.001 by ANOVA. C) Alteration in averaged tumor volumes in different experimental groups of mice (*n* = 6 for each group). Data are presented as mean ± SEM. *Compared with control group, *P < 0.05, **P < 0.01, ***P < 0.001 by ANOVA; ^#^Compared with U0126 group, ^###^ *P* < 0.001 by ANOVA. D) Typical picture of excised tumor in each group. scale bar: 1 cm. E) Averaged tumor weights of different experimental groups of mice at week 5 after cancer cell injection. Data are presented as mean ± SEM. *Compared with control group, *P < 0.05, **P < 0.01, ***P < 0.001 by ANOVA. F) The mRNA and protein expression levels of B‐RAF in patients with pancreatic adenocarcinoma. Data are presented as mean ± SEM. **P < 0.01 by T‐test. G) A proposed model for the cross‐talk between WNT and B‐RAF signaling pathways. (Related Figure S7 and Table S1, Supporting Information).

Mutations in *RNF43* gene activate the WNT pathway in cancer cells. Here, we found that *RNF43*‐mutated cancer cells exhibited increased MEK activity. Therefore, we examined the synergism between WNT and MEK inhibitors. The combination of LGK974 (a WNT pathway inhibitor) and U0126 showed a synergistic effect in the inhibition of proliferation and colony formation in *RNF43*‐mutated AsPC‐1 and HPAF‐II cells, but not in PANC‐1 cells (**Figure** **6**A,B). Consistent with the results for pancreatic cancer, *RNF43*‐mutated SW48 colon cancer cells were more sensitive to the three MEK inhibitors than CaCO2 cells expressing wild‐type *RNF43* (Figure S7A, Supporting Information). In addition, synergism between the WNT and MEK inhibitors was observed only in SW48 cells (Figure S7B, Supporting Information). Similarly, *RNF43*‐mutated UACC‐257 melanoma cells showed higher sensitivity to MEK inhibitors than Mel1617 cells expressing wild‐type *RNF43* (Figure S7C, Supporting Information). Synergism of the inhibition combination was observed in UACC‐257 cells, but not in Mel1617 cells (Figure S7D, Supporting Information). These data suggest that the combination of WNT and MEK inhibitors suppresses *RNF43*‐mutated cancer cells more effectively. To confirm treatment efficiency in vivo, nude mice were subcutaneously injected with AsPC‐1 cells and treated with the solvent, U0126, LGK974, or both. Reduced tumor sizes and weights were found in mice treated with the combination of U0126 and LGK974 compared to the control groups (Figure 6C–E), which is consistent with our findings in cultured cancer cells. In addition, analysis of pancreatic adenocarcinoma patients in The Cancer Genome Atlas (TCGA) database showed similar B‐RAF mRNA levels among the patients, but dramatically high B‐RAF protein levels in patients with mutated *RNF43* (Figure 6F), supporting our hypothesis that RNF43 controls B‐RAF abundance via post‐translational regulation.

In conclusion, we identified B‐RAF as an in vivo substrate of RNF43 and revealed the underlying mechanism through which RNF43 negatively regulates the B‐RAF/MEK pathway (Figure 6G).

## 3 Discussion

Our understanding of the physiological and pathological roles of RNF43 is still in an early stage, as the mechanism of regulation of its ligase activity remains unclear, and only a few in vivo substrates of RNF43 have been identified to date. Variations or modifications at specific sites in RNF43 protein can affect its function. The C‐terminal truncations identified within the D504‐Q563 region of RNF43, as revealed by the cBioPortal database, switch RNF43 to an oncogenic protein by trapping casein kinase 1 to prevent β‐catenin turnover to enhance ligand‐independent activation.^[^ ^16^ ^]^ In contrast, mutations in AsPC‐1 (RNF43‐S720X) and HPAF‐II (RNF43‐E174X) have been confirmed to result in truncated and non‐functional RNF43 proteins.^[^ ^17^ ^]^ Tsukiyama et al. demonstrated that RNF43 activity is controlled by phosphorylation of three serine residues by casein kinase 1.^[^ ^18^ ^]^ Substitution of the serine trio with alanine abolished the activity of RNF43, triggering FZD degradation and inhibiting WNT signaling. However, the phospho‐deficient mutant behaved similar to the phospho‐mimicking mutant in terms of FZD binding, protein dimerization, and intracellular localization. These results suggest that phosphorylation enhances RNF43‐mediated ubiquitination; however, whether phosphorylation affects the conformation of the RING domain of RNF43 to increase the accessibility of FZD proteins for ubiquitination remains unknown. In this study, we identified B‐RAF as an in vivo substrate of RNF43 and elucidated the role of RNF43 in modulating the activation of the MEK/ERK signaling pathway through B‐RAF in pancreatic cancer cells. Decreased levels of another candidate, PAK5, were observed in cells overexpressing RNF43. Although no interaction between PAK5 and RNF43 was detected, RNF43KO PANC‐1 cells with depleted B‐RAF or PAK5 showed considerably reduced colony‐forming abilities (Figure S2B, Supporting Information) compared to PANC‐1 cells, indicating that RNF43 may regulate the proliferation of pancreatic cancer cells via B‐RAF or PAK5. Therefore, further investigation is warranted.

Crosstalk between the WNT and ERK pathways has been proposed previously. GSK3β, a component of the β‐catenin destruction complex, phosphorylates RAS proteins and triggers β‐transducin repeat‐containing protein recruitment to promote RAS degradation and ERK inactivation.^[^ ^19^ ^]^ β‐catenin protein stabilized by WNT activation or adenomatous polyposis coli (APC) loss in turn binds to the RAS protein to prevent its phosphorylation by GSK3β and enforces ERK signaling.^[^ ^20^ ^]^ Conversely, a recent study has shown that increased WNT signaling suppresses ERK activity and ERK‐mediated gene expression.^[^ ^21^ ^]^ Moreover, Zhan et al. found that truncated APC mutations in colorectal cancer strongly synergize with MEK inhibitors to enhance WNT responses.^[^ ^22^ ^]^ When this study was ongoing, a clinical investigation reported the association between RNF43 mutations and anti‐BRAF/EGFR therapies in BRAF^V600E^ colorectal cancer patients, suggesting cross‐talk between the WNT and MAPK pathways and supporting the findings of this study.^[^ ^23^ ^]^ Our results demonstrate that B‐RAF is a direct ubiquitination target of RNF43, adding a new level of interplay between these two signaling pathways.

B‐RAF has been reported to be ubiquitinated by several E3 ligases.^[^ ^24^ ^]^ The major biological consequence of B‐RAF ubiquitination by these E3 ligases is proteasomal degradation, whereas K27‐linked ubiquitination induces B‐RAF activation.^[^ ^24e^ ^]^ Our data showed that RNF43 ubiquitinates B‐RAF on K499 to trigger B‐RAF proteolysis. Notably, the ectopic expression of RNF43 also downregulated the protein levels of the B‐RAF V600E mutant in UACC‐257 melanoma cells and the oncogenic B‐RAF deletion mutant in BxPC‐3 pancreatic cancer cells (Figure S1, Supporting Information).^[^ ^25^ ^]^ Several studies have demonstrated that the application of proteolysis‐targeting chimeras (PROTACs) to trigger B‐RAF protein degradation has effective anti‐cancer activity.^[^ ^26^ ^]^ Our data demonstrated that RNF43 targets wild‐type and mutant B‐RAF for degradation, and restoration of this E3 ligase may overcome B‐RAF inhibitor resistance. Since RNF43 is an endogenous cellular protein, the ectopic expression of this ligase may prevent PROTAC‐induced uncontrollable protein downregulation and off‐target effects.

We found that RNF43‐mediated B‐RAF ubiquitination at K499 was negatively regulated via T491 phosphorylation. Three clinically identified small deletions (ΔNVTAP, ΔTAPTP, and ΔPTPQQ) close to or containing T491 in B‐RAF, reported in pancreatic and thyroid cancers, also alter the structure and activation of B‐RAF.^[^ ^27^ ^]^ Dynamic phosphorylation of T491 may be a critical mechanism for controlling the stability of B‐RAF under various physiological and pathological conditions. Our data also suggested that ERK is a potential kinase that phosphorylates T491 in B‐RAF (Figure 4J,K). However, T491 may be phosphorylated by multiple kinases in vivo. Consequently, additional analysis is warranted.

Aberrant activation of the WNT pathway is frequently observed in human cancers. Chemical inhibitors or biologics targeting WNT signaling molecules, such as FZD receptors, β‐catenin, and porcupine are under development.^[^ ^28^ ^]^ Clinical trials for the treatment of cancer patients with porcupine inhibitors are ongoing, and RNF43 mutations are being defined as predictive biomarkers for patient selection.^[^ ^29^ ^]^ However, porcupine inhibitors exhibit several off‐target side effects.^[^ ^30^ ^]^ Therefore, a combination of different inhibitors may be an alternative strategy. A previous study by Steinhart et al. demonstrated the potential of WNT‐FZD5 signaling as a therapeutic target in *RNF43*‐mutant pancreatic cancers. Substantial responses were observed in both in vitro and in vivo models following treatments with anti‐FZD5 antibodies.^[^ ^17^ ^]^ Our findings suggest that a combination of WNT and MEK inhibitors may be advantageous for the treatment of RNF43‐deficient PDAC. Therefore, a prospective experimental approach may involve the combination of anti‐FZD5 antibodies and MEK inhibitors for PDAC treatment.

## 4 Conclusion

In summary, this study is the first to demonstrate that B‐RAF is an in vivo substrate of RNF43. Moreover, we revealed the mechanism underlying negative regulation of the B‐RAF/MEK pathway in pancreatic cancer cells and animal models. Based on this finding, we have additional insights into the progression of pancreatic cancer.

## 5 Experimental Section

### Cell Culture

PANC‐1, Caco‐2, SW48, and HEK293T cells were cultured in Dulbecco's Modified Eagle Medium (DMEM, Hyclone, GE Healthcare Life Sciences, Logan, AP, USA) containing 10% fetal bovine serum (FBS) (GIBCO, Thermo Fisher Scientific, Waltham, MA, USA) and 1% penicillin/streptomycin (PS) (GIBCO). AsPC‐1, BxPC‐3, Mel1617, and UACC‐257 cells were cultured in RPMI‐1640 medium (GIBCO) supplied with 10% FBS and 1% PS. HPAF‐II cells were cultured in Eagle's Minimum Essential Medium (MEM, Corning, Mamassas, VA, USA) containing 10% FBS and 1% PS. HPDE cells were grown in keratinocyte serum‐free media (GIBCO) supplemented with 1% PS, 5 ng mL^−1^ human EGF and 50 µg mL^−1^ bovine pituitary extract (BPE). hTERT‐HPNE cells were cultured in medium containing 75% DMEM without glucose (Sigma‐Aldrich, Co., St. Louis, MO, USA), 2 mm L‐glutamine, 1.5 mg mL^−1^ sodium bicarbonate, 25% M3: BaseF (INCELL Co., San Antonio, TX, USA), 5% FBS, 10 ng mL^−1^ rEGF, 5.5 mm D‐glucose, and 750 ng mL^−1^ puromycin. PANC‐1, AsPC‐1, HPAF‐II, CaCO2, SW48, and HEK293T cells were purchased from the American Type Culture Collection (Manassas, VA, USA). Mel1617 and UACC‐257 cells were kindly provided by Dr. Che‐Hung Shen (National Health Research Institutes). BxPC‐3 cells were kindly provided by Dr. Kuang‐Hung Cheng (National Sun Yat‐sen University). HPDE cells were kindly provided by Dr. Wun‐Shaing Wayne Chang (National Health Research Institutes). The hTERT‐HPNE cells were kindly provided by Dr. Hua‐Kuo Tai (National Taiwan University). Cell line identities were verified by short tandem repeat analysis and were confirmed as Mycoplasma free. PANC‐1 cells with stable expressions of pLHCX (vector only), pLHCX‐B‐RAF‐wt, pLHCX‐B‐RAF‐K499R, and pLHCX‐B‐RAF‐T491A were generated by electroporation delivery and maintained in the DMEM medium supplemented with 50 µg mL^−1^ hygromycin B, 10% FBS, and 1% PS.

### Kinase Screening

Cells were seeded at 3 × 10^3^ cells per well in 96‐well plates and separately treated with 160 kinase inhibitors (Cayman Chemical, MI, USA) at 100 nm. After 72 h, the medium was replaced with 100 µL culturing medium containing 10 µL of CellTiter 96 AQueous One Solution reagent (Promega, Madison, WI, USA). Cells were incubated for 3 h under 5% CO_2_ at 37 °C and analyzed for OD^490^ using the TECAN Sunrise ELISA Reader (Tecan Group Ltd., Männedorf, Switzerland.). IC_50_ value resulting from 50% inhibition of cell growth was calculated graphically as a comparison with control group. All analyzed kinases and screening results are listed in Table S5 (Supporting Information).

### Western Blotting

Cell lines were lysed in radioimmunoprecipitation assay (RIPA) buffer (50 mm Tris‐HCl, pH 8.0, with 150 mm sodium chloride, 1.0% Igepal CA‐630 (NP‐40), 0.5% sodium deoxycholate, and 0.1% sodium dodecyl sulfate) with fresh‐added Halt Protease & Phosphatase Single‐Use Inhibitor Cocktail (PI; Thermo Fisher Scientific, Waltham, MA, USA). Mouse tumors were lysed in RIPA buffer containing PI and 100 µg mL^−1^ proteinase K. Proteins were separated on sodium dodecyl sulfate‐polyacrylamide gel electrophoresis (SDS‐PAGE) and transferred onto polyvinylidene fluoride membranes (Immobilon‐P, Merck Millipore, Billerica, MA, USA). The membranes were blocked with 5% milk, incubated with indicated primary antibodies at 4 °C overnight, and incubated with suitable secondary antibodies at room temperature for 1 h. Immunoreactivities of the membranes were detected with the Western Lightning Plus‐ECL Enhanced Chemiluminescence Substrate (PerkinElmer, Inc., Waltham, MA, USA). At least three experiments were performed to confirm the results.

### shRNA Transfection and Transient Knockdown

The shRNA plasmids were purchased from the National RNAi Core Facility (Academia Sinica, Taipei, Taiwan). hTERT‐HPNE cells were transfected with the plasmid containing shLuc or shRNF43 using Xfect transfection reagent (Takara Bio., Kusatsu, Japan). After transfection for 48 h, the transfected cells were collected for further analysis.

### CRISPR/Cas9 Mediated Gene Editing in RNF43

Plasmids containing sgRNA targeting RNF43 (RNF43‐KO plasmids, sgRNA sequence: GATCCTCAGTGATGTCAAAG) were obtained from the National RNAi Core Facility (Academia Sinica, Taipei, Taiwan). The RNF43‐KO plasmids were co‐transfected with pMD.G and pCMVΔR8.91 (Academia Sinica, Taipei, Taiwan) into HEK293T cells for generating virus. After 72 h, the medium was collected and condensed with Lenti‐X concentrator (TAKARA, CA, USA). For targeting RNF43 in PANC‐1 and HPDE cells, 20 µl virus medium and 8 µg mL^−1^ polybrene were added to the cells seeded in 12‐well plates (1.5 × 10^6^ cells per well). After 24 h, the cells were re‐seeded into 10 cm dishes and selected with puromycin (2 µg mL^−1^ for PANC‐1 and 0.5 µg mL^−1^ for HPDE cells). After 7d, the cells were plated as single cells on 96‐well plates. RNF43 protein expression levels in RNF43‐KO PANC‐1 and HPDE cells were analyzed using western blotting.

### Plasmid Construction

Human RNF43 Gene ORF cDNA clone expression plasmid, pCMV3‐SP‐Myc‐RNF43, was purchased from Sino Biological Inc., Beijing, PRC. Human B‐RAF Gene ORF cDNA clone expression plasmid, pLHCX‐Flag‐B‐RAF, was kindly provided by Dr. Che‐Hung Shen. Site direct mutagenesis was performed using the QuickChange Site‐Directed Mutagenesis Kit (Stratagene, Agilent Technologies, Palo Alto, CA, USA) according to the manufacturer's recommended protocol. The primer pairs for constructing various mutants are listed in Table S4 (Supporting Information).

### Ubiquitination Assay

AsPC‐1, HPAF‐II, PANC‐1, and HEK293T cells were transfected with indicated plasmids for 24 h. MG132 (30 µM) was added for 3 h. The cells were collected and lysed in RIPA buffer containing PI and 5 mm N‐Ethylmaleimide to prevent degradation of ubiquitinated proteins. The ubiquitinated proteins were immunoprecipitated with indicated antibodies and analyzed with western blotting.

### Immunoprecipitation Assay

For analysis of co‐immunoprecipitation, cells were lysed in RIPA buffer (50 mm Tris‐HCl, pH 8.0, with 150 mm sodium chloride, 1.0% Igepal CA‐630 (NP‐40), 0.5% sodium deoxycholate, and 0.1% sodium dodecyl sulfate (SDS)) containing PI and 5 mm N‐Ethylmaleimide (Sigma‐Aldrich, Co., St. Louis, MO, USA). For analysis of ubiquitinated‐B‐RAF, cells were lysed in modified RIPA buffer with 1% SDS, boiled at 80 °C for 10m, and diluted with RIPA buffer. The lysate was incubated with indicated antibodies at 4 °C overnight. Protein A/G Mag Sepharose Xtra (GE Healthcare Bio‐Sciences AB, Uppsala, Sweden) were subsequently added. After incubation at 4 °C for 4 h, the immunoprecipitates were washed and boiled in sample buffer and analyzed with Western blotting.

### Mass Spectrometry Analysis

Flag‐B‐RAF protein was purified from HEK293 cells overexpressing Flag‐B‐RAF, Flag‐B‐RAF plus myc‐RNF43, or Flag‐B‐RAF plus myc‐mutated RNF43 with Anti‐FLAG M2 Magnetic Beads (Merck Millipore, Billerica, MA, USA). The immunoprecipitated protein complexes were separated by using SDS‐PAGE. The SDS‐PAGE was incubated sequentially in fixing solution (50% methanol and 10% glacial acetic acid) at room temperature for 1 h, in staining solution (0.1% Coomassie Brilliant Blue R250, 50% methanol and 10% glacial acetic acid) at room temperature for 1 h, and in destaining solution (40% methanol and 10% glacial acetic acid). The SDS‐PAGE was cut into pieces according to the protein size. The gels were digested with trypsin and analyzed by mass spectrometry using Thermo Scientific Orbitrap Elite (OmicsLab Co., Ltd., Taipei, Taiwan). The results were further analyzed by using the MASCOT software (Version 2.6.0) and the SwissProt database (2017_03; 553 941 sequences; 198 311 666 residues).

### Clonogenic Assay

For comparison of various B‐RAF mutations, PANC‐1 cells were transfected with indicated plasmids. After 24 h, the cells were reseeded at 500 cells per well into 6‐well plates. For comparison of various treatments, AsPC‐1, HPAF‐II, and PANC‐1 cells were treated with U0126 (2 µM), LGK974 (2 µM), or both. The cells were allowed to grow for 7–14 d. Colonies were visualized by methylene blue staining and counted under a microscope.

### Structure Manipulation and Binding Affinity Prediction

T491 phosphorylation in B‐RAF was manipulated by using the software package AMBER 18 with the Amber additional force fields from the AMBER database (http://research.bmh.manchester.ac.uk/bryce/amber#pro).^[^ ^31^ ^]^ The 3D structure of full‐length RNF43 was generated by homology modelling with I‐TASSER webserver (https://zhanglab.ccmb.med.umich.edu/I‐TASSER/). Then the full‐length RNF43 structure was aligned with the reference structure (PDB ID: 5TTE) to generate the complex structures, including B‐RAF (with or without Thr^491^ phosphorylation), RNF43, and ubiquitin proteins. The complex structures were inserted into TIP3P solvent molecules. These initial complexes were then simulated using AMBER 18 with the Amber99sB and Amber additional force fields. All MD simulations were performed in the isothermal–isobaric (NPT) ensembles with a simulation temperature of 310 K, unless otherwise stated, using the Verlet integrator with an integration time step of 0.002 ps and SHAKE constraints for all covalent bonds involving hydrogen atoms.^[^ ^32^ ^]^ In the electrostatic interactions, atom‐based truncation was performed using the PME method,^[^ ^33^ ^]^ and the switch van der Waals function was used with a 2.00‐nm cutoff for atom‐pair lists. These complex structures were minimized for 100000 conjugate gradient steps and then subjected to 10‐ns NPT MD simulations. The final structures from these simulations were used to initiate the binding energies calculations. The binding energies between B‐RAF (with or without T491 phosphorylation) and RNF43 or ubiquitin were calculated by using PRODIGY (https://bianca.science.uu.nl//prodigy/).^[^ ^34^ ^]^

### Animal Xenograft Model

For comparison of various B‐RAF mutations, tumor cell‐based xenografts were established by subcutaneous injection of 5 × 10^6^ PANC‐1 derivatives (PANC‐1‐ctrl, PANC‐1‐B‐RAF‐wt, PANC‐1‐B‐RAF‐K499R, PANC‐1‐B‐RAF‐T491A; *n* = 5 for each group) into the right flanks of Advanced Severe Immuno Deficiency (ASID) mice. All animal experiments were approved by the Animal Care Committee of National Health Research Institutes (approval No. 112011). Tumors were measured with digital calipers weekly. Tumor volumes were calculated according to the formula V  =  (long axis × short axis^2^)/2. All animals were maintained in specific pathogen‐free conditions with water and feed ad libitum. Eight week after the injection, the tumors were harvested and tumor weight was measured. For comparison of various treatments, 3 × 10^6^ AsPC‐1 cells were injected subcutaneously into the right flanks of ASID mice. Tumors were measured with digital calipers twice a week. Two weeks after inoculation, mice were randomly divided into groups of 6 mice each, with equal tumor size distribution (average and variance). Solvent (control), U0126 (0.5 mg kg^−1^), LGK974 (5 mg kg^−1^) or both was administered daily through intraperitoneal injection. After a 3‐w period of treatment, the tumors were harvested and tumor weight was measured.

### Immunohistochemical (IHC) Staining

Paraffin‐embedded sections were de‐waxed, rehydrated, and blocked for endogenous peroxidase and nonspecific binding sites. Tissue sections were stained with indicated antibodies overnight at 4 °C followed by incubation with horseradish peroxidase (HRP)‐conjugated secondary antibodies for 1 h at room temperature. The protein signal was developed using a 3,3′‐diaminobenzidine (DAB) solution. After counterstaining with hematoxylin and being sealed, images of the IHC‐stained slides were captured using a Carl Zeiss Axioskop 2 plus microscope (Carl Zeiss, Thornwood, NY, USA) and analyzed using ImageJ software.^[^ ^35^ ^]^

### Drug Combination Analysis

Cells were treated with U0126 or LGK974 for 72 h to determine the concentration that induced a 50% inhibition of cellular growth (IC50) in cell viability assay. U0126 was combined with LGK974 at a constant ratio determined by IC50 _U0126_/IC50 _LGK974_. The effects of drug combinations were evaluated with SiCoDEA (https://sicodea.shinyapps.io/shiny/) and Calcusyn software (Biosoft, Cambridge, UK) according to Chou–Talalay combination index method.^[^ ^36^ ^]^ CI > 1 indicates antagonism, CI = 1 indicates additive effect, and CI < 1 indicates synergism. All experiments were performed in triplicate.

### Senescence Assay

hTERT‐HPNE cells were transfected with control plasmid pcDNA3.1(‐) or pcDNA3.1(‐)‐KRAS‐G12D combined with plasmid containing shLuc or shRNF43 using Xfect transfection reagent (Takara Bio., Kusatsu, Japan). After 48 h, the transfected hTERT‐HPNE cells were fixed for senescence analysis by using senescence β‐galactosidase staining kit (Cell Signaling Technology Inc., Beverly, MA, USA) according to the manufacturer's protocol.

### Clinical Database Analysis

Data were collected from the cBioPortal (https://www.cbioportal.org/), the Catalogue Of Somatic Mutations In Cancer (COSMIC, https://cancer.sanger.ac.uk/cosmic), and the Human Protein Atlas (https://www.proteinatlas.org).

### Statistical Analysis

All experiments were performed at least three times. Data were normalized to the control group in each experiment. Statistical Package for Social Sciences version 16.0 (SPSS 16.0; SPSS Inc.) was applied to perform statistical analyses. Statistical differences between two groups were assessed by independent samples *t*‐test. For comparisons among more than two groups, statistical differences were assessed by one‐way analysis of variance (ANOVA) with LSD post hoc test. Values were presented as mean ± SEM. *P* < 0.05 was considered significant.

## Conflict of Interest

The authors declare no conflict of interest.

## Ethics approval statement

This manuscript is original, has not been previously published, and will not be submitted elsewhere for publication. The submission has received explicit approval from all co‐authors. Authors whose names appear on the submission have contributed sufficiently to the scientific work and therefore share collective responsibility and accountability for the results. The present study followed international, national, and/or institutional guidelines for animal treatments and complied with relevant legislation from the Animal Care Committee of National Health Research Institutes (ethical approval No. 112011).

## Supporting information

Supporting Information

## Data Availability

The clinical results that support the findings of this study were extracted from the cBioPortal (https://www.cbioportal.org), the Catalogue Of Somatic Mutations In Cancer (COSMIC, https://cancer.sanger.ac.uk/cosmic), and the Human Protein Atlas (https://www.proteinatlas.org).
