# Supplementary material for: RNF43 Inactivation Enhances the B‐RAF/MEK Signaling and Creates a Combinatory Therapeutic Target in Cancer Cells
Source: Adv Sci (Weinh). 2024 Jan 15;11(12):2304820. doi: 10.1002/advs.202304820 (PMC10966525; doi:10.1002/advs.202304820)
Supplement: Supplementary file 1 — Supporting Information [file ADVS-11-2304820-s001.pdf]

ADVANCED SCIENCE
Open Access

Supporting Information

for *Adv. Sci.*, DOI 10.1002/advs.202304820

RNF43 Inactivation Enhances the B-RAF/MEK Signaling and Creates a Combinatory Therapeutic Target in Cancer Cells

*Shih-Han Hsu, Ya-Li Tsai, Yeng-Tseng Wang, Che-Hung Shen, Yu-Hsuan Hung, Li-Tzong Chen and Wen-Chun Hung**

ADVANCED SCIENCE Open Access

Supporting Information

for *Adv. Sci.*

RNF43 Inactivation Enhances the B-RAF/MEK Signaling and Creates a Combinatory Therapeutic Target in Cancer Cells

*Shih-Han Hsu, Ya-Li Tsai, Yeng-Tseng Wang, Che-Hung Shen, Yu-Hsuan Hung, Li-Tzong Chen, and Wen-Chun Hung**

RNF43 Inactivation Enhances the B-RAF/MEK Signaling and Creates a Combinatory Therapeutic Target in Cancer Cells

Shih-Han Hsu[1], Ya-Li Tsai[1], Yeng-Tseng Wang[2], Che-Hung Shen[1], Yu-Hsuan Hung[1], Li-Tzong Chen[1,3,4], and Wen-Chun Hung[1,5,6,*]

[1]National Institute of Cancer Research, National Health Research Institutes, Tainan 704, Taiwan

[2]Department of Biochemistry, College of Medicine, Kaohsiung Medical University, Kaohsiung 804, Taiwan

[3]Division of Hematology & Oncology, Department of Internal Medicine, Kaohsiung Medical University Hospital, Kaohsiung 804, Taiwan

[4]Faculty of Medicine, College of Medicine, Kaohsiung Medical University, Kaohsiung 807, Taiwan

[5]Department of Pharmacy, College of Pharmacy, Kaohsiung Medical University Hospital, Kaohsiung 807, Taiwan

[6]Department of Biological Science and Technology, National Yang Ming Chiao Tong University, Hsinchu 300, Taiwan

*Corresponding author. Email: hung1228@nhri.edu.tw.

Supporting information includes 7 Supplementary Figures and 5 Supplementary Tables.

Supplementary Figures

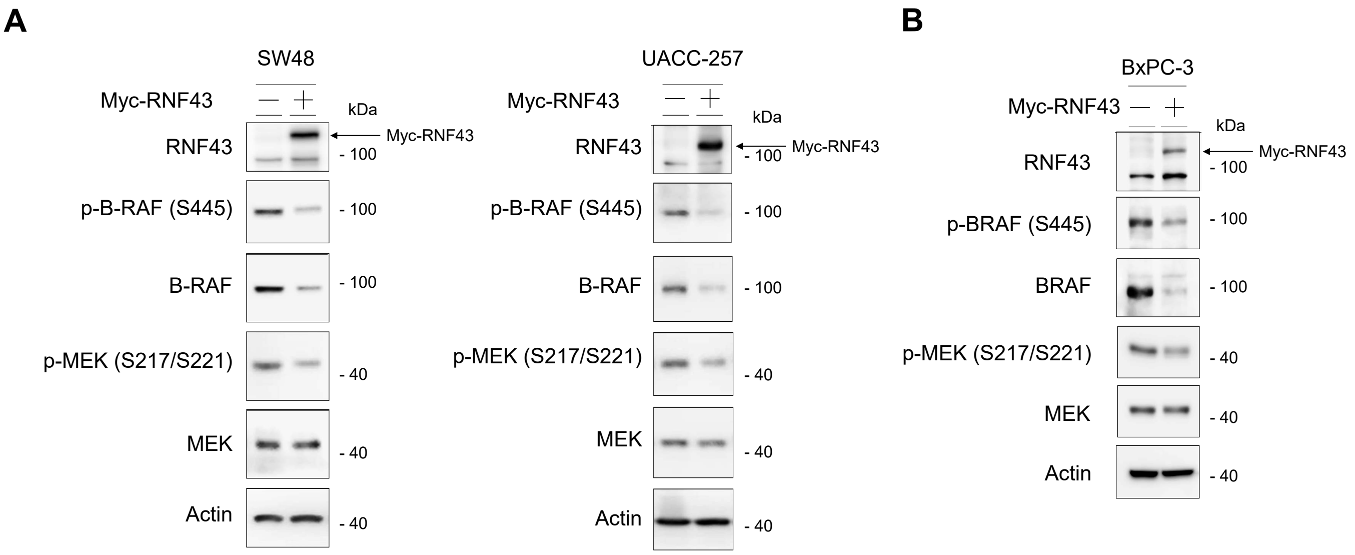

Figure S1.
Overexpression of wild-type RNF43 in cancer cells expressing mutated *RNF43* or mutated *B-RAF* reduces the B-RAF expression and MEK activity.
SW48 colon cancer cells (A, mutated-*RNF43* and wild-type-*B-RAF*), UACC-257 melanoma cells (A, mutated-*RNF43* and mutated-*B-RAF*), and BxPC-3 pancreatic cancer cells (B, mutated-*RNF43* and mutated-*B-RAF*) were transfected with Myc-RNF43 expression vector. After 24 h, cells were collected for Western blot analysis.

Related to Figure 1 and Table S1

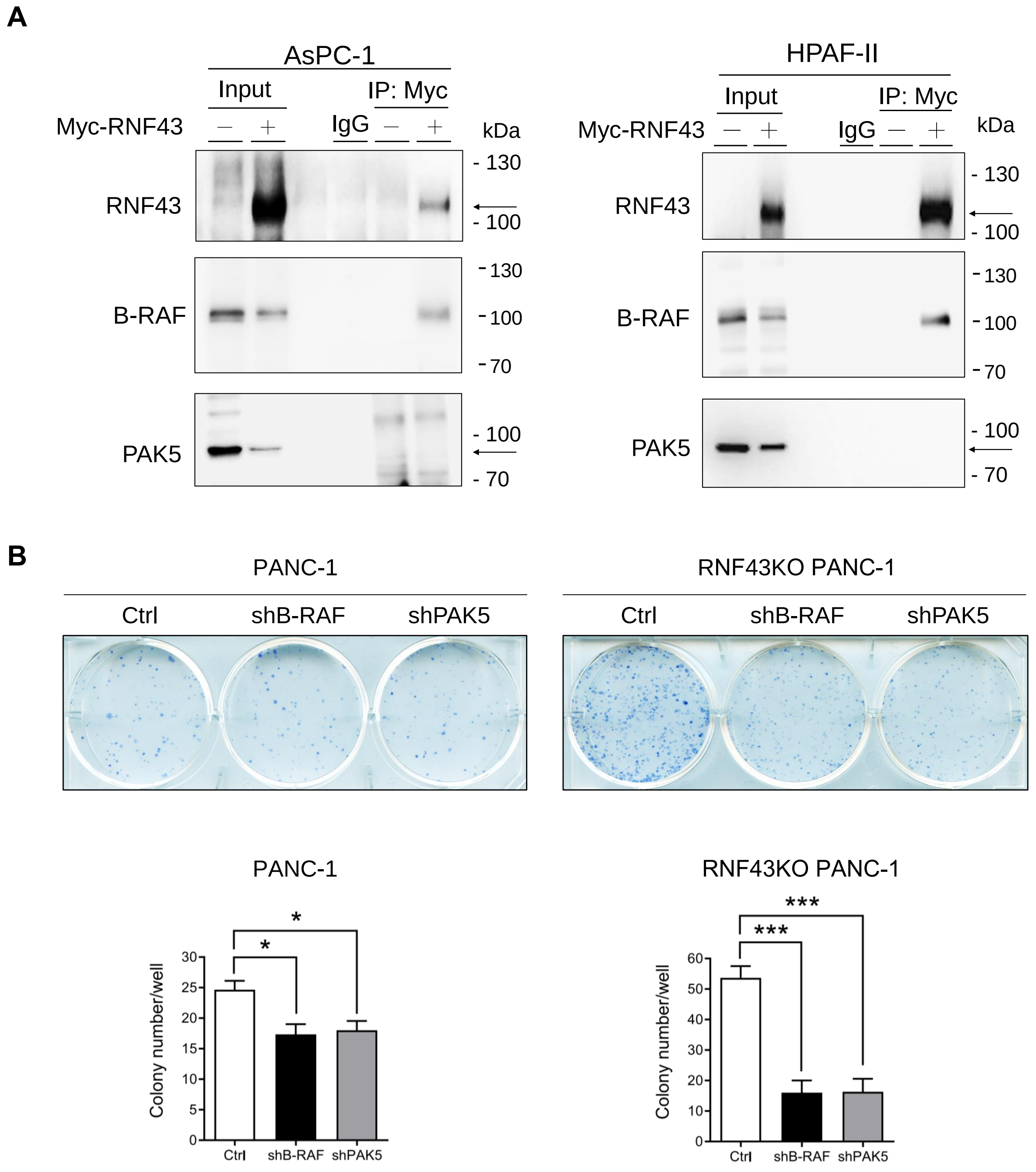

Figure S2.

Interactions and regulations of B-RAF and PAK5 by RNF43 in pancreatic cancer cells.

(A) AsPC-1 and HPAF-II cells were transfected with or without Myc-RNF43 and immunoprecipitated with beads coating RNF43 antibody, Myc antibody, or control IgG. Western blot was performed using antibodies against RNF43, B-RAF, and PAK5. Considerable interaction between RNF43 and B-RAF, but not PAK5, was observed in AsPC-1 and HPAF-II cells.

(B) Colony-forming abilities of PANC-1 and RNF43KO PANC-1 cells with depleted B-RAF or PAK5.

Related to Figure 1 and 2

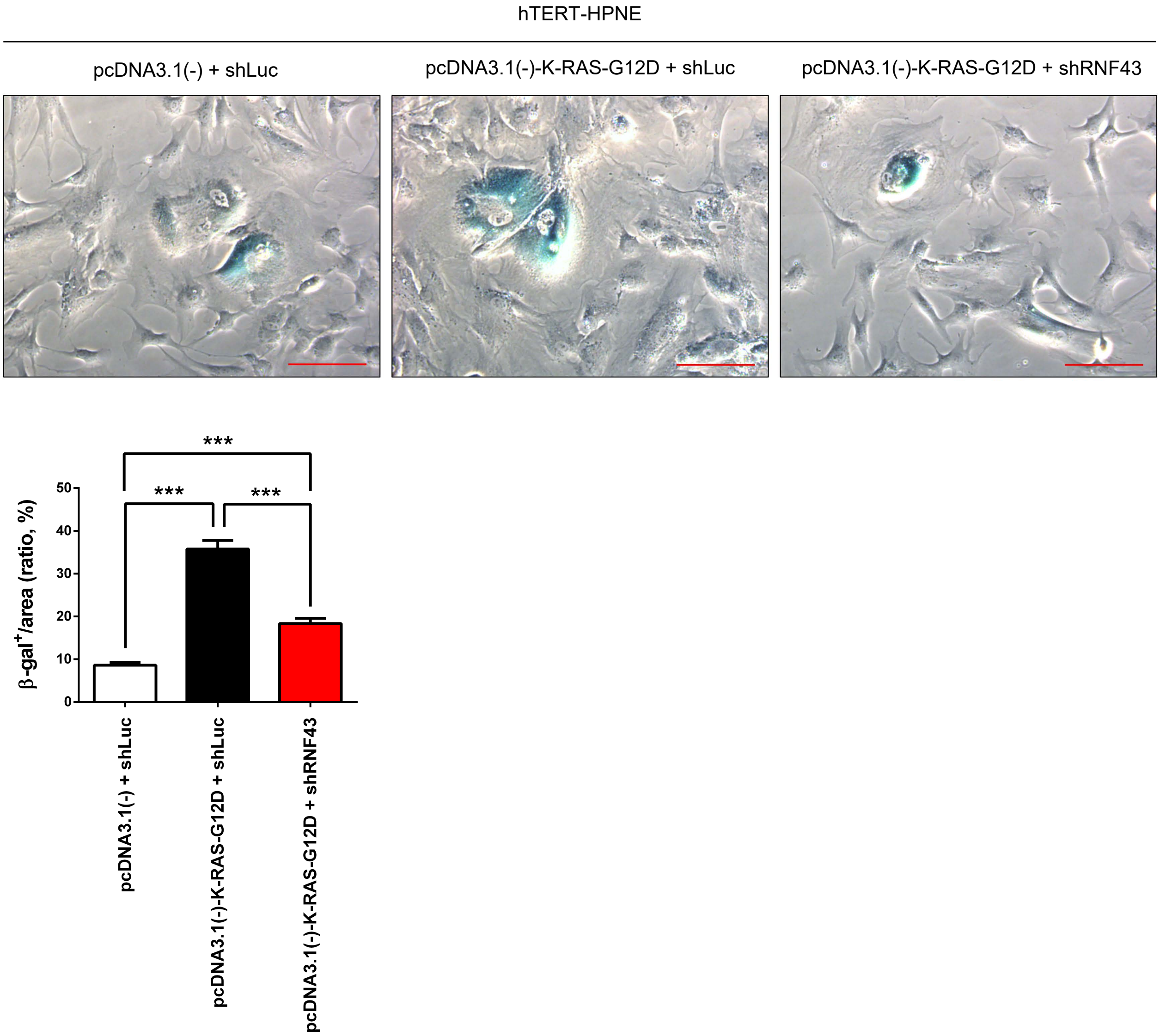

Figure S3.

Depletion of RNF43 rescues mutant K-RAS (G12D)-induced senescence.

The hTERT-HPNE cells were co-transfected with indicated combination of plasmids. The senescence status was examined by β-galactosidase staining. Scale bar: 100 μm. Data are presented as mean ± SEM (n = 25). ****P* < 0.001 by ANOVA.

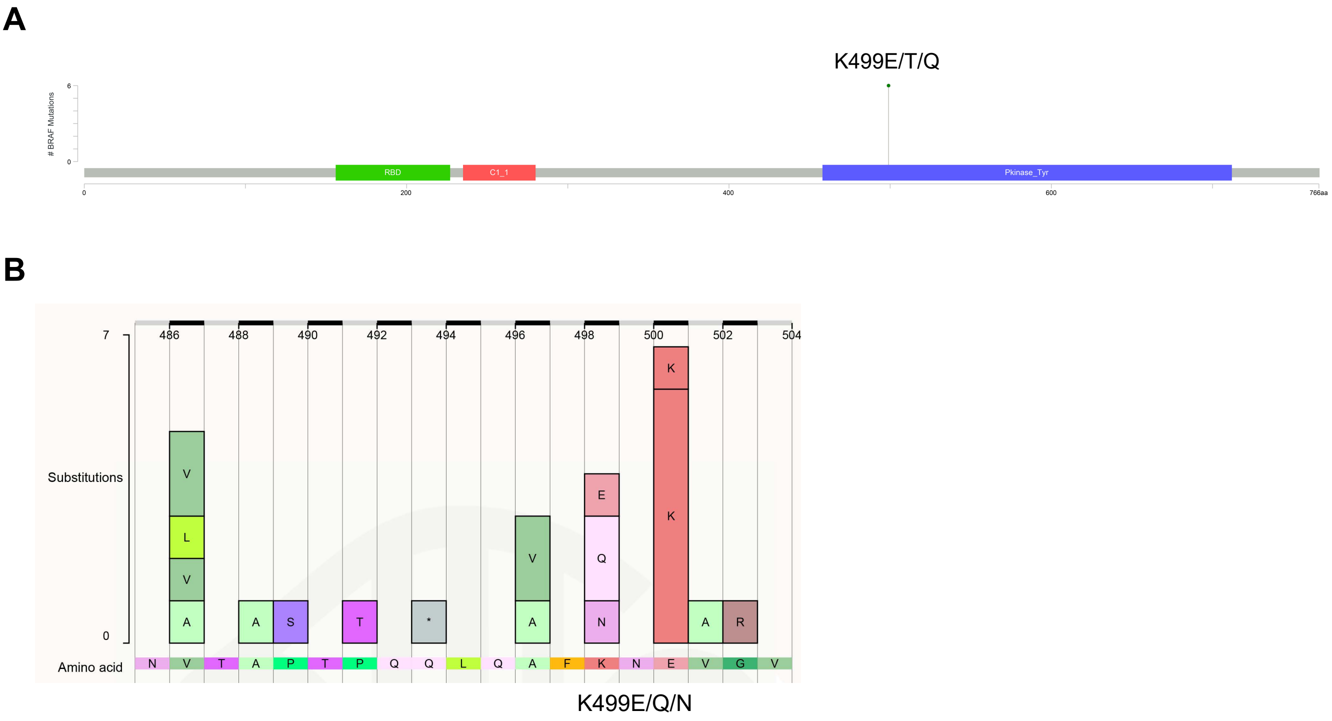

Figure S4.
Mutations at K499 in B-RAF in cancer patients.
Analysis of the online database including cBioPortal (A) and COSMIC (B) revealed several pathogenic K499 mutations (K499E, K499N, K499Q, and K499T) in B-RAF. RBD: Raf-like Ras-binding domain (156-227). C1_1: Phorbol esters/ diacylglycerol binding domain (C1 domain) (235-280). Pkinase_Tyr: Protein tyrosine kinase (458-712).

Related to Figure 3 and Table S2

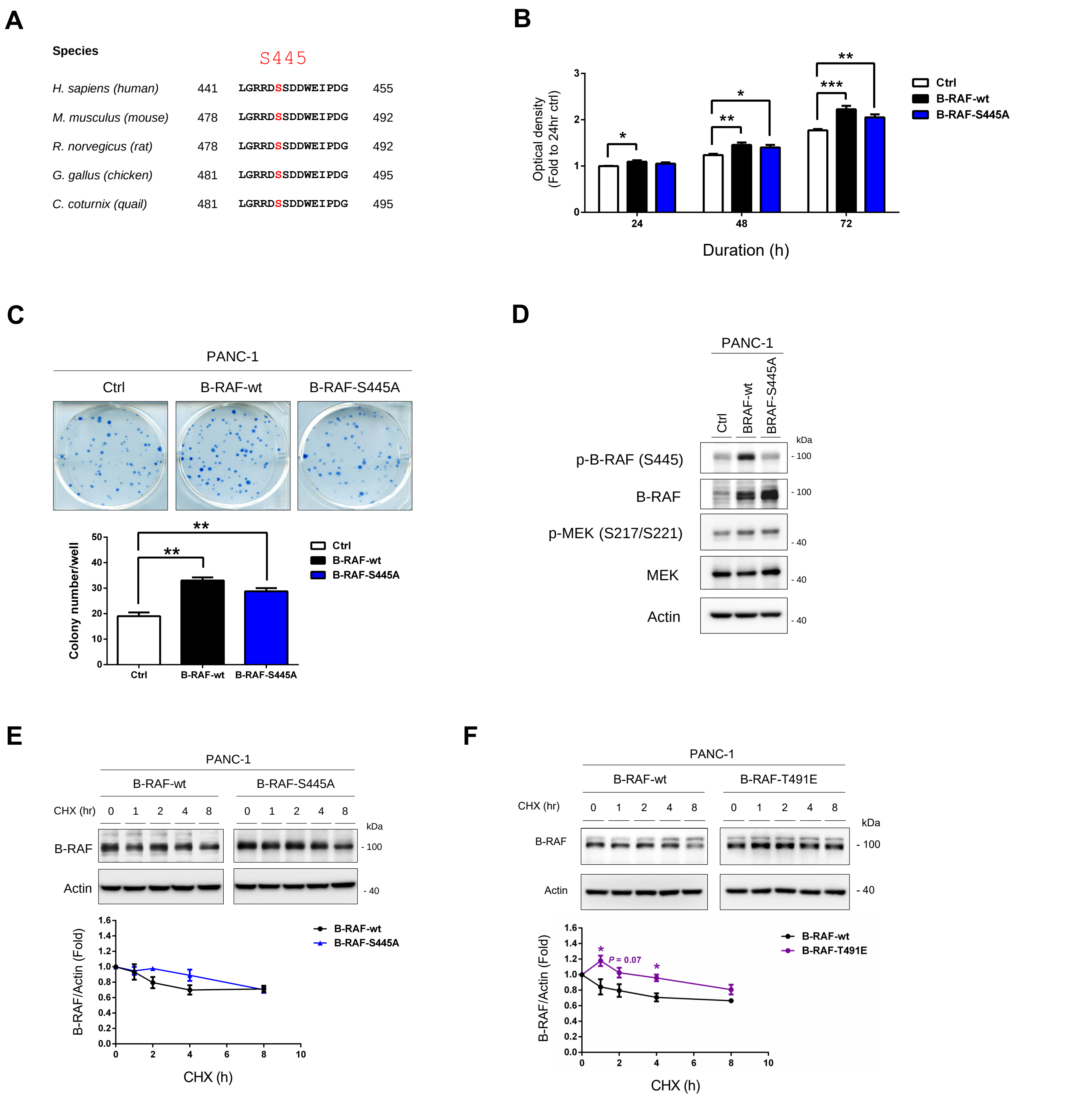

Figure S5.

S445A mutation in B-RAF does not significantly affect B-RAF stability or proliferation in PANC-1 cells.

(A) Alignment of the 441-455 region of human B-RAF with various species.

(B, C, and D) PANC-1 cells were transfected with control, wild-type B-RAF, or S445A mutant vectors. Cellular proliferations (B, n = 12) and the numbers and sizes of colonies (C, n = 4) were measured. Data are presented as mean ± SEM. $*P < 0.05$, $**P < 0.01$, $***P < 0.001$ by ANOVA. The protein levels and activities of indicated proteins (D) in the cells were studied.

(E) PANC-1 cells transfected with wild-type B-RAF or S445A mutant vectors were treated with cycloheximide. Protein stabilities of wild-type B-RAF and S445A mutant were compared (n = 4). Data are presented as mean ± SEM.

(F) PANC-1 cells transfected with wild-type B-RAF or T491E mutant vectors were treated with cycloheximide. Protein stabilities of wild-type B-RAF and T491E mutant were compared (n = 4). Data are presented as mean ± SEM. $*P < 0.05$ by T-test.

Related to Figure 4 and Table S3

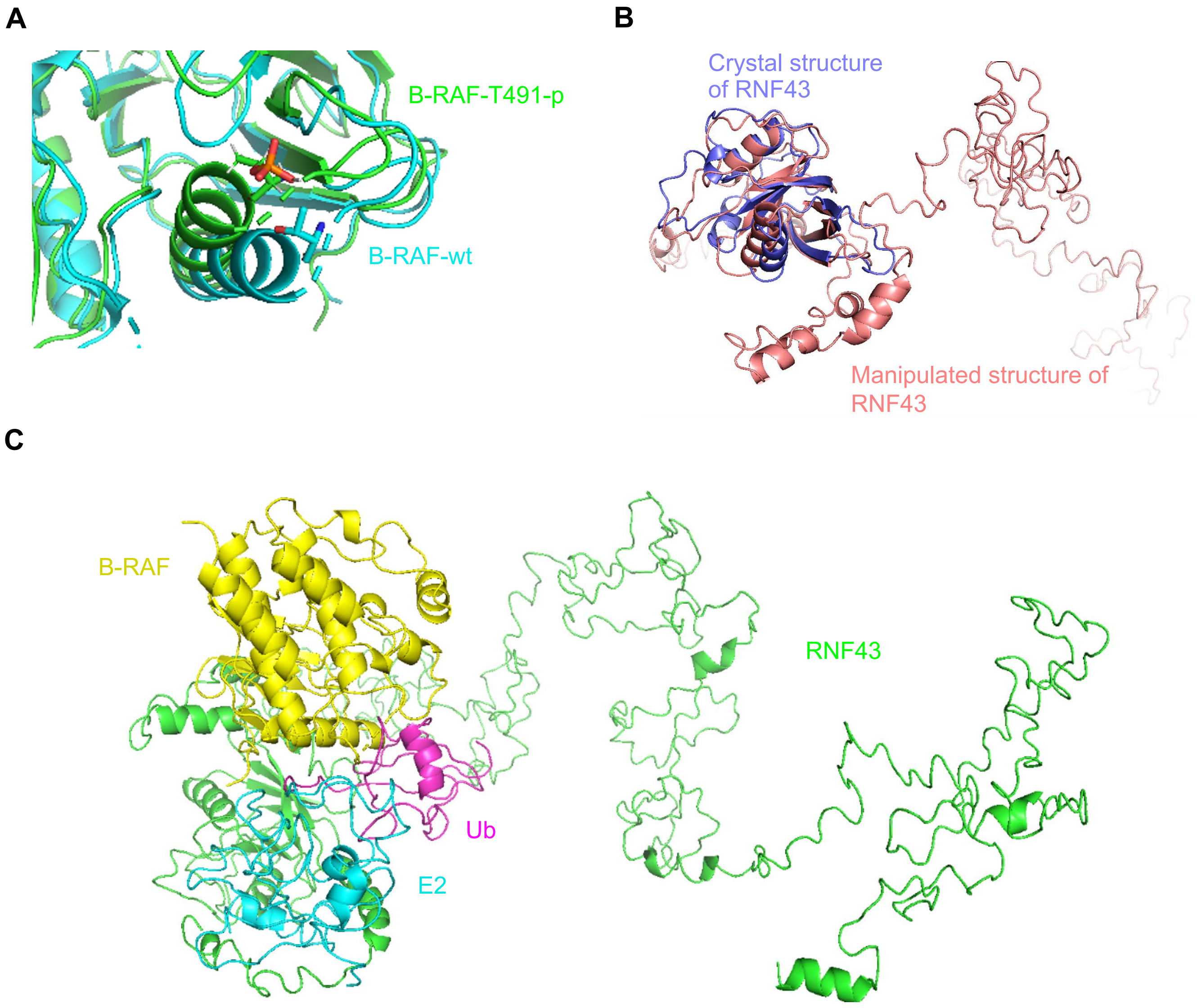

Figure S6.
T491 phosphorylation leads to a conformational change in B-RAF.
(A) Comparison of the structure of 485-503 region in B-RAF (cyan) and B-RAF phosphorylated at T491 position (light green).
(B) Full-length model construction of RNF43. The structure colored in purple was a reported RNF43 crystal structure (PDB ID: 4KNG). The structure colored in light red was the 3D structure of full-length RNF43 generated by homology modelling with I-TASSER webserver. Alignment of the two structures showed high similarity of the RNF43 model (light red) and the known structure of RNF43 fragment (purple).
(C) Simulation of the protein-protein interactions among B-RAF (yellow), RNF43 (light green), ubiquitin (Ub, pink) and E2 (cyan). The E3 ligase ARIH1 in a reported crystal structure of protein complex containing B-RAF, ubiquitin, E2 ligase, and ARIH1 (PDB ID: 5TTE) was replaced by the full-length RNF43 model.

Related to Figure 4 and Table S3

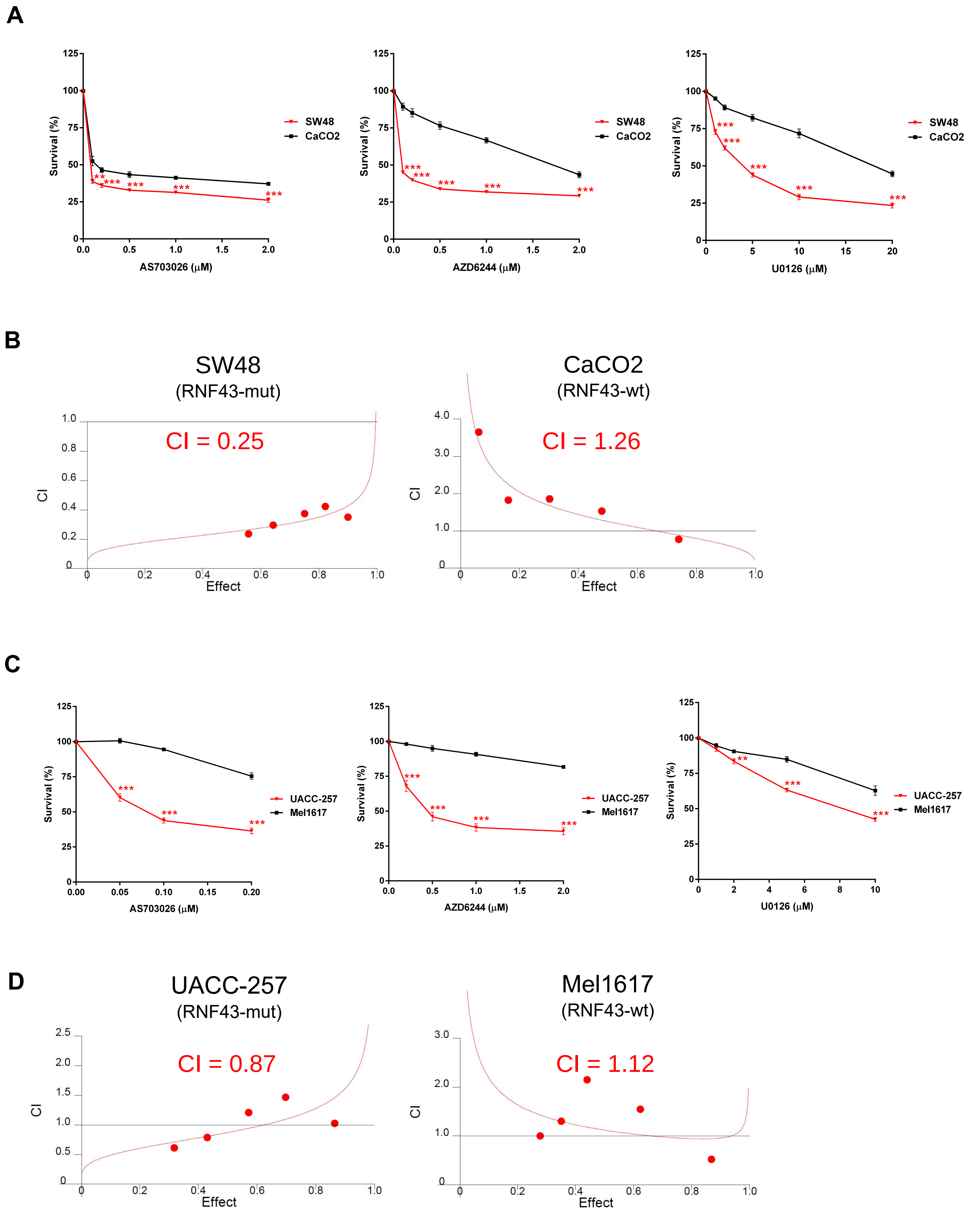

Figure S7.

Drug sensitivities and drug combinations in colorectal carcinoma cells and melanoma cells.

(A) Sensitivity to MEK inhibitors AS703026, AZD6244, and U0126 in RNF43-mutated SW48 and RNF43-wild-type CaCO2 colorectal cancer cells (n = 9). Data are presented as mean ± SEM. **$P < 0.01$, ***$P < 0.001$ by T-test.

(B) CI values calculated from combination of U0126 and LGK974 in SW48 and CaCO2 cells (n = 9).

(C) Sensitivity to MEK inhibitors in RNF43-mutated UACC-257 and RNF43-wild-type Mel1617 melanoma cells (n = 9). Data are presented as mean ± SEM. **$P < 0.01$, ***$P < 0.001$ by T-test.

(D) CI values calculated from combination of U0126 and LGK974 in UACC-257 cells and Mel1617 cells (n = 9).

Related to Figure 6 and Table S1

Supplementary Tables

Table S1. Sensitivity of pancreatic cancer, melanoma, and colorectal cancer cell lines to MEK inhibitors including AS703026, AZD6244, and U0126. (n = 9 for each cell line)

Drug IC50 (μM, mean ± SEM)	Pancreatic cancer			Colorectal cancer		Melanoma	
	AsPC-1 (Mutated *RNF43*)	HPAF-II (Mutated *RNF43*)	PANC-1 (Wild type *RNF43*)	SW48 (Mutated *RNF43*)	CaCO2 (Wild type *RNF43*)	UACC-257 (Mutated *RNF43*)	Mel1617 (Wild type *RNF43*)
AS703026	0.10 ± 0.001	0.13 ± 0.02	5.68 ± 1.28	0.08 ± 0.002	0.17 ± 0.03	0.08 ± 0.01	0.40 ± 0.01
AZD6244	0.53 ± 0.04	0.47 ± 0.08	20.83 ± 1.84	0.09 ± 0.001	1.76 ± 0.04	0.52 ± 0.09	4.36 ± 0.12
U0126	9.41 ± 0.90	9.15 ± 0.36	25.97 ± 1.26	4.01 ± 0.25	17.82 ± 0.84	8.18 ± 0.27	13.25 ± 0.74

(Related to Figure 1, 6, S1, and S2)

Table S2. Datasets with K499 mutations in B-RAF from cBioPortal.

Study of Origin	Sample ID	Protein Change	Cancer Type Detailed
Melanoma (MSKCC, 2018)	P-0039410-T01-IM6	K499E	Cutaneous Melanoma
MSK-IMPACT Clinical Sequencing Cohort (MSKCC, Nat Med 2017)	P-0009086-T01-IM5	K499E	Upper Tract Urothelial Carcinoma
China Pan-cancer (OrigiMed2020)	P-2435	K499E	Gastric Adenocarcinoma
Colorectal Adenocarcinoma (DFCI, Cell Reports 2016)	coadread_dfci_2016_2969	K499Q	Colorectal Adenocarcinoma
Cancer Cell Line Encyclopedia (Novartis/Broad, Nature 2012)	JHUEM7_ENDOMETRIUM	K499T	Endometrial Carcinoma
Cancer Cell Line Encyclopedia (Broad, 2019)	JHUEM7_ENDOMETRIUM	K499T	Endometrial Carcinoma

(Related to Figure 3 and S4)

Table S3. Prediction of binding energy (kcal/mol) between indicated proteins.

	RNF43	Ubiquitin
B-RAF-T491 (wild type)	-9.0	-12.3
B-RAF-T491-p (phosphorylated)	-7.0	-7.2

(Related to Figure 4, S5, and S6)

Table S4. Primer sets for site direct mutagenesis in this study.

Primer sets		Sequence
RNF43-C290S	Forward primer	5′-TACGGGTCATTTCCTCCCTCCATGAGTTCCA-3′
	Reverse primer	5′-TGGAACTCATGGAGGGAGGAAATGACCCGTA-3′
RNF43-C298S	Forward primer	5′-AGTTCCATCGTAACTCTGTGGACCCCTGGTT-3′
	Reverse primer	5′-AACCAGGGGTCCACAGAGTTACGATGGAACT-3′
B-RAF-K499R	Forward primer	5′-AGCAGTTACAAGCCTTCCGAAATGAAGTAGGAGTAC-3′
	Reverse primer	5′-GTACTCCTACTTCATTTCGGAAGGCTTGTAACTGCT-3′
B-RAF-T491A	Forward primer	5′-GAATGTGACAGCACCTGCACCTCAGCAGTTACA-3′
	Reverse primer	5′-TGTAACTGCTGAGGTGCAGGTGCTGTCACATTC-3′
B-RAF-T491E	Forward primer	5′-ATGTGACAGCACCTGAACCTCAGCAGTTACA-3′
	Reverse primer	5′-TGTAACTGCTGAGGTTCAGGTGCTGTCACAT-3′

Table S5. Kinase screening in this study.

	Inhibition rate (n=3, mean)		
	AsPC-1	HPAF-II	PANC-1
1 A5 Paclitaxel 10461	63%	49%	38%
1 H7 Staurosporine 81590	55%	34%	19%
1 C5 INK128 11811 (mTORC1/2 inhibitor)	47%	40%	-6%
1 B7 AS-703026 11226 (MEK 1/2 inhibitor)	34%	35%	-9%
1 A6 Erlotinib 10483 (tyrosine kinase inhibitor which acts on the epidermal growth factor receptor (EGFR)	32%	30%	7%
1 C10 PD 0325901* 13034 (MEK inhibitor)	25%	24%	-12%
2 A10 JNJ-10198409 10008131	21%	42%	29%
2 B7 PIK-75 (hydrochloride) 10009210	17%	18%	-5%
1 A8 NVP-BEZ235 10565 (PI3K and mTOR inhibitor)	17%	32%	2%
2 A11 Leelamine (hydrochloride) 10008614	12%	19%	11%
1 B10 AZD 7762 11491	12%	26%	-11%
1 G7 PP242 13643	11%	12%	-10%
1 A4 Doramapimod 10460	11%	28%	9%
2 B11 (R)-Roscovitine 10009569	11%	3%	-28%
2 A3 Leelamine 10006148	11%	19%	-2%
2 A6 TGX-221 10007349	10%	22%	-1%
1 C6 Canertinib (hydrochloride) 12076	9%	-3%	-15%
1 A10 AG-879 10793	9%	26%	-4%
2 B2 Arachidonic Acid Leelamide 10008617	9%	-2%	6%
1 A2 TG003 10398	8%	29%	5%

2 A2 Y-27632 (hydrochloride) 10005583	8%	15%	5%
2 H4 PD 184161* 10012431	7%	20%	15%
1 A9 Phthalazinone pyrazole 10735	6%	22%	2%
2 A9 D-erythro-Sphingosine C-18 10007907	6%	18%	0%
1 A11 1-NA-PP1 10954	6%	25%	-2%
2 A8 AS-605240 10007707	6%	18%	10%
2 D2 AG-494 10010242	6%	6%	-15%
2 H3 CAY10578 10011264	5%	15%	-10%
2 A5 PD 169316 10006727	5%	16%	5%
2 H2 CAY10577 10011256	5%	10%	14%
2 A4 PD 98059 10006726	4%	16%	1%
2 C2 BAY 43-9006 10009644	4%	8%	4%
2 A7 (S)-H-1152 (hydrochloride) 10007653	4%	18%	-7%
1 A3 PKC 412 10459	4%	31%	13%
2 G2 AG-370 10010568	3%	3%	-20%
2 F2 Lavendustin C 10010329	2%	7%	0%
2 G3 Wortmannin 10010591	1%	3%	8%
2 E2 AG-18 10010300	1%	7%	-26%
2 C11 Olomoucine 10010240	1%	-1%	-10%
1 H2 SU 6668 13873	1%	24%	9%
1 C7 SB-431542 13031	1%	-15%	-18%
2 G11 NSC 210902 10011255	0%	4%	-7%

2 H5 CCT018159 10012591	0%	11%	-11%
1 H10 PD 166326 9000988	-1%	28%	1%
2 B3 Lauric Acid Leelamide 10008618	-1%	-10%	-12%
2 C3 CAY10561 10010043	-2%	-4%	-12%
2 G4 AG-1296 10010592	-2%	-6%	-18%
1 B8 Chelerythrine (chloride) 11314	-2%	15%	-14%
2 E11 AG-183 10010315	-2%	0%	-22%
2 B9 Piceatannol 10009366	-2%	5%	-16%
2 B8 Sphingosine Kinase Inhibitor 2 10009222	-3%	10%	-2%
2 G5 KT 5823 10010965	-3%	-8%	-18%
1 H11 O-1918 10004914	-3%	18%	0%
1 H6 U-0126 70970	-3%	31%	2%
1 H3 PHA-767491 18218	-3%	25%	11%
1 G11 CAY10626 13838	-4%	34%	4%
2 B10 SC-1 10009557	-4%	25%	-9%
2 B5 CAY10505 10009078	-4%	-4%	-13%
2 D11 Apigenin 10010275	-4%	5%	-36%
1 B11 GSK1059615 11569	-4%	20%	-9%
1 H8 KN-92 (hydrochloride) 9000890	-4%	23%	2%
1 B3 BIBF 1120 11022	-4%	26%	2%
2 F3 ZM 336372 10010367	-5%	-23%	-15%
1 B2 Torin 1 10997	-5%	22%	-2%

2 G7 CAY10574 10011247	-5%	-7%	3%
1 B5 Bisindolylmaleimide XI (hydrochloride) 11073	-5%	17%	-15%
2 B6 PI-103 10009209	-5%	11%	-11%
1 H9 AS-605240 (potassium salt) 9000980	-5%	20%	0%
2 G6 Janex 1 10011246	-5%	4%	0%
2 C9 Erbstatin Analog 10010238	-5%	-7%	-19%
2 D3 AG-825 10010243	-5%	-4%	-14%
1 E11 KN-62 13318	-5%	16%	-12%
1 H5 LY294002 70920	-5%	25%	5%
2 F4 5-Iodotubercidin 10010375	-6%	-5%	-14%
1 B9 Tunicamycin 11445	-6%	17%	-9%
2 F11 HA-1077 (hydrochloride) 10010559	-6%	6%	-7%
1 D2 VX-702 13108	-6%	23%	-8%
2 C5 PI3-Kinase α Inhibitor 2 10010177	-6%	-7%	-10%
1 G2 CAY10621 13371	-6%	14%	-8%
1 H4 N,N-Dimethylsphingosine 62575	-6%	25%	5%
1 G3 YM-201636 13576	-7%	3%	-6%
2 E3 CAY10554 10010301	-7%	0%	-17%
1 B4 SMI-4a 11029	-7%	18%	-13%
2 C8 Triciribine 10010237	-7%	13%	-11%
1 E2 Bisindolylmaleimide IV 13299	-7%	12%	-8%
1 F11 SB 203580 (hydrochloride) 13344	-7%	15%	-14%

2 G8 CAY10576 10011249	-8%	4%	-7%
1 G6 NVP-AEW541 (hydrochloride) 13641	-8%	17%	-11%
1 G4 ZM 447439 13601	-8%	8%	-13%
2 B4 AS-252424 10009052	-8%	-9%	-3%
1 C11 SB 203580 13067	-8%	16%	-10%
1 D11 Bisindolylmaleimide I 13298	-8%	15%	-12%
1 B6 CAY10657 11140	-9%	13%	-16%
1 C2 Ruxolitinib 11609	-9%	18%	-6%
2 G9 NH125 10011250	-9%	-11%	2%
2 C10 Kenpaullone 10010239	-9%	-14%	-23%
1 G8 ABT-869 13653	-9%	-2%	-16%
1 G10 17β-hydroxy Wortmannin 13812	-10%	9%	-17%
2 D9 LFM-A13 10010265	-10%	-15%	-37%
1 F2 KN-93 13319	-10%	11%	-4%
2 F5 SB 202190 10010399	-10%	-15%	-10%
2 F6 CAY10571 10010400	-10%	4%	1%
1 F7 Bisindolylmaleimide IX (mesylate) 13334	-10%	-14%	-13%
1 G9 CAY10622 13687	-10%	-4%	-21%
2 G10 TWS119 10011251	-10%	6%	-1%
1 D7 Sunitinib (malate)* 13159	-11%	-27%	-19%
2 D4 AG-1478 10010244	-11%	0%	-21%
2 C7 ML-9 10010236	-11%	0%	-21%

2 C6 CAY10567 10010233	-11%	2%	-22%
2 D8 H-8 (hydrochloride) 10010249	-11%	0%	-31%
1 E7 Gö 6983 13311	-12%	-26%	-15%
2 E4 DRB 10010302	-12%	-7%	-19%
1 G5 AS-041164 13622	-12%	1%	-17%
2 F7 Nilotinib 10010422	-12%	-24%	-8%
2 F8 SP 600125 10010466	-12%	-3%	-12%
1 C4 SB-505124 11793	-12%	-15%	-18%
2 F9 L-threo-Sphingosine C-18 10010541	-12%	-21%	-19%
2 E5 RG-13022 10010309	-13%	-23%	-13%
1 D5 BIO 13123	-13%	-20%	-17%
1 C8 PD 173074* 13032	-13%	-7%	-13%
2 C4 AS-604850 10010175	-13%	-6%	-9%
2 F10 H-89 10010556	-14%	-11%	-6%
2 E9 AG-99 10010313	-14%	-13%	-22%
2 D5 SB-216763 10010246	-14%	-24%	-19%
2 D7 AG-17 10010248	-14%	6%	-38%
2 D10 SC-514 10010267	-14%	-17%	-15%
2 E7 AG-490 10010311	-14%	-9%	-30%
2 E6 RG-14620 10010310	-15%	-7%	-14%
2 D6 SB-415286 10010247	-15%	-5%	-19%
1 D4 CHIR99021 13122	-16%	-16%	-21%

2 E8 AG-82 10010312	-16%	0%	-18%
1 E10 NU 6102 13317	-16%	-23%	-16%
2 E10 AG-213 10010314	-16%	-21%	-9%
1 D6 Imatinib (mesylate) 13139	-16%	-21%	-21%
1 D10 3-Methyladenine 13242	-17%	-19%	-17%
1 C9 Valproic Acid (sodium salt) 13033	-17%	-11%	-19%
1 F10 LY364947 13341	-18%	-14%	-23%
1 E4 NSC 663284 13303	-18%	-11%	-17%
1 C3 Necrostatin-1 11658	-19%	-7%	-16%
1 F4 Iso-Olomoucine 13325	-19%	-15%	-17%
1 F8 ST638 13337	-19%	-22%	-7%
1 F9 SU6656 13338	-20%	-16%	-21%
1 E8 H-9 (hydrochloride) 13312	-20%	-23%	-10%
1 E5 D 4476 13305	-20%	-26%	-19%
1 E9 Indirubin-3’-monoxime 13314	-20%	-23%	-18%
1 F5 (S)-Glycyl-H-1152 (hydrochloride) 13332	-20%	-24%	-19%
1 D8 Gefitinib 13166	-20%	-17%	-11%
1 F3 CGP 57380 13322	-20%	-15%	-13%
1 E3 Bisindolylmaleimide V 13300	-21%	-17%	-15%
1 D9 PP2 13198	-23%	-14%	-19%
1 E6 NU 7026 13308	-23%	-28%	-19%
1 D3 Emodin 13109	-24%	-11%	-22%

1 F6 Bisindolylmaleimide VIII (acetate) 13333	-24%	-14%	-16%
